# Association Between Dietary Live Microbes and Diet Quality Among Children and Adults in France

**DOI:** 10.1111/nbu.70057

**Published:** 2026-05-23

**Authors:** Rozenn Gazan, Matthieu Maillot, Florent Vieux, Romane Poinsot, Adam Drewnowski

**Affiliations:** ^1^ MS‐Nutrition, Faculté de Médecine la Timone, Laboratoire C2VN Marseille France; ^2^ Center for Public Health Nutrition University of Washington Seattle Washington USA

**Keywords:** diet quality, dietary assessment, fermented foods, France, live microbes, microbiota, National Dietary Survey, socioeconomic factors

## Abstract

Consumption of foods containing live microbes has been associated with multiple health benefits. This study assessed exposure to live microbes in foods, including fermented foods, in the nationally representative INCA 3 dietary survey (2014–2015) in France. Foods in the French nutrient composition database (CIQUAL) were assigned to low, medium or high food groups of live microbe content. Dietary intakes came from the last French national dietary survey (INCA3 2014–2015). Participants were stratified by socio‐demographic characteristics and by tertiles of medium/high food consumption. Diet quality measures were mean adequacy ratio (MAR, %), mean excess ratio (MER, %) and a score developed by the Programme National Nutrition Santé (sPNNS‐GS2). Foods with Medium/High levels of live microbes were consumed by 97.7% of children and 98.4% of adults. Yogurt and cheese (unpasteurised) were the top sources of high food codes (99%). Vegetables and fruit (in particular, raw and unpeeled) were the top sources of medium food codes (75%). The leading fermented foods consumed in France were yogurt and cheese. Consumption of medium/high foods was 166.8 g/day for children and 215.9 g/day for adults. Medium/High foods accounted for < 4.5% of daily energy but provided > 10% of some key nutrients. Consumption of foods containing medium/high levels of live microbes was associated with higher socio‐economic status and with higher quality diets. This was the first analysis of exposure to dietary live microbes in France by population strata.

## 1 Introduction

Foods containing live microbes are a continuing topic of research interest. Live microbes, a part of the food microbiome, are thought to interact with the gut microbiota, immune system, and metabolic pathways to benefit gut health (Wiertsema et al. 2021; Berg et al. 2025). Fermented foods and minimally processed fresh produce are the chief sources of live microbes (Berg et al. 2025; Rezac et al. 2018). Fermented dairy foods include yogurt (with active cultures), kefir and raw milk cheeses such as Camembert or Brie. Fermented vegetable products include sauerkraut (unpasteurised), kimchi and fermented pickles with live cultures. Fermented soy products include miso, natto and tempeh. When not pasteurised, fermented foods often contain lactic acid bacteria, yeasts or other beneficial microbes (Rezac et al. 2018).

Fresh produce can also be a source of live microbes. Potential sources include leafy greens (lettuce, spinach and kale), root vegetables (carrots and radishes), cruciferous vegetables (broccoli and cabbage), berries and fresh produce from local farms. These fresh foods can carry environmental bacteria from the soil, water and air, microbes from the plant leaf surface, and lactic acid bacteria (Berg et al. 2025; Marco et al. 2020; Rezac et al. 2018). The microbes are generally non‐pathogenic when the vegetables are fresh, washed properly and from a safe source. Washing and peeling reduce microbial load (Mendoza et al. 2022).

Live microbes are thought to have numerous health benefits such as improved digestive function (Shi et al. 2024; Yang et al. 2024), greater gut microbiome diversity (Leeuwendaal et al. 2022) and improved gastrointestinal symptoms (Mukherjee et al. 2025). There is some evidence for reduced gut inflammation (Balasubramanian et al. 2024), better cardiometabolic health (Hill et al. 2023) and even less anxiety, depression and stress (Balasubramanian et al. 2024; Shi et al. 2024). There is a health claim authorised for use in the EU and GB ‘live cultures in yoghurt or fermented milk improve lactose digestion of the product in individuals who have difficulty digesting lactose’ (EFSA Panel on Dietetic Products, Nutrition and Allergies (NDA) 2010). In order to bear the claim, yogurt or fermented milk should contain at least 10^8^ Colony Forming Units live starter microorganisms ( *Lactobacillus delbrueckii* subsp. bulgaricus and *Streptococcus thermophilus* ) per gram (DHSC 2020; European Commission 2026). There is also growing evidence suggesting that cheese, a product of fermentation, is not associated with adverse cardiometabolic outcomes and may even exert protective effects (Eugénio et al. 2025).

Exposure to dietary live microbes in the US was recently examined (Marco et al. 2022) using data from the National Health and Nutrition Examination Survey (NHANES 2001–2018). Foods consumed by NHANES participants were assigned into three classes based on presumed levels of live microbe content: Low (< 104 CFU/g), Medium (104–107 CFU/g) or High (> 107 CFU/g). About 59% of children and 67% of adults consumed foods in the Medium/High classes (Marco et al. 2022). Per capita intake of Medium/High foods was 85 g/d for children and 127 g/day for adults.

The consumption of vegetables, salads and dairy products including yogurt and cheese is known to be much higher in France than in the US (Tamers et al. 2009; Franzon et al. 2025). Hence the motivation to explore population exposure to foods containing live microbes in relation to diet quality in France. This is the first analysis of exposure to foods containing live microbes in relation to diet quality using the latest French Individual and National Food Consumption Survey (INCA3).

## 2 Methods

### 2.1 The INCA 3 Population Survey

Dietary intakes data came from the third French Individual and National Food Consumption Survey (INCA3), conducted between February 2014 and September 2015 by the French Agency for Food, Environmental and Occupational Health & Safety (Agence nationale de sécurité sanitaire de l'alimentation de l'environnement et du travail [ANSES]) (ANSES 2019). This cross‐sectional survey was based on two independent representative samples of children (0–17 years) and adults (18–79 years) residing in mainland France (Dubuisson et al. 2019). The survey design of INCA3 used three‐level random sampling: geographic primary units (PUs), followed by households (secondary units) within the PUs, and lastly individuals (tertiary units) within the households. After the exclusion of young children (< 4 years), the final INCA 3 sample was 2121 adults (≥ 18 years) and 1775 children (≥ 4 years).

Socio‐demographic data were sex, age class, socio‐professional category (SPC), income per consumption unit (CU) and body mass index (BMI). Age groups for adults were 18–44 years, 45–64 years and > 65 years. Following the International Standard Classification of Occupations (International Labour Office 2012), SPC was divided into low (mainly office and manual workers), medium (mainly craftspeople, company directors/owners and other intermediate professions) and high (mainly executives and self‐employed professionals), and a fourth class, labelled as not working, including retired, used to work, students and non‐working spouses. Income per CU was categorised as < 900 €/month/CU, 900–1339 €/month/CU, 1340–1849 €/month/CU, ≥ 1850 €/month/CU. Bodyweight and height were measured during home visits. BMI values for adults were categorised as < 18, 18–25, 25–30, 30–35 and > 35 kg/m^2^. Obesity in children aged 2–17 years was defined based on criteria established by the International Obesity Task Force (Cole et al. 2000). These criteria correspond to the BMI percentile curves of the reference population, which reach the cutoff values of 18.5 kg/m^2^, 25 kg/m^2^ and 30 kg/m^2^ at age 18.

### 2.2 Dietary Intakes From INCA 3 Study

Dietary intakes in the INCA 3 survey were collected by telephone. Interviews for both children and adults were conducted by professional interviewers trained on the updated French version of GloboDiet software (formerly EPIC‐Soft) (Slimani et al. 2011). Participants aged 15–79 years self‐reported their food and beverage intakes for 2 or 3 non‐consecutive 24‐h periods over 3 weeks, including two weekdays and one weekend day. The days were not announced in advance to avoid bias. Participants aged < 15 years were provided with 24 h open‐ended food records to be completed on pre‐determined 3 non‐consecutive days. Data collection days were determined at the time of the home visit by the investigator. A follow‐up call was made the day before and the 24 h dietary recall was most often conducted the next day, or at the latest 3 days later. Children could be assisted by caregivers or other persons responsible for their nutrition.

Participants were asked to report all foods and beverages consumed during the day or at night during the preceding 24 h by eating occasion. A list of 19 possible descriptors was used to describe as precisely as possible the food or beverage consumed (e.g., skimmed, low fat, reduced fat or whole milk). All foods declared in the survey were kept in the analyses. Food portion sizes were estimated using a picture book of food servings, household measures (Trolle et al. 2013; Van Kappel et al. 1995) or were directly expressed by weight/volume or standard units. Nutrients intakes are provided for each individual and each eating occasion. The present analyses were based on three 24 h dietary recalls.

### 2.3 Classification of Foods by Low, Medium and High Microbe Content

Foods in the ANSES/French Information Centre on Food Quality (CIQUAL) database (ANSES 2016a) excluding alcoholic beverages were categorised into 11 food groups and 24 subgroups as shown in Table 1 and Table S1. In a US study, Marco et al. (2022) used a categorisation into three classes to represent the live microbe content in Colony Forming Unit (CFU). Marco et al. classified, based on expertise, 9388 Food and Nutrient Database for Dietary Studies (FNDDS) food codes into Low (< 104 CFU/g), Medium (104–107 CFU/g) and High (> 107 CFU/g) classes. These classes were chosen to reflect the approximate numbers of viable microbes expected to be present in pasteurised foods (< 104 CFU/g), fresh fruits and vegetables eaten unpeeled (104–107 CFU/g), and unpasteurised fermented foods and probiotic supplements (> 107 CFU/g). As many as 8954 foods in the FNDDS database were assigned to the low class (95.4% of all foods).

**TABLE 1:** Percentage of foods by Low, Medium or High live microbe content by sub‐subgroup (subgroups with 100% low in Table S1).

Food groups	Food subgroup	Food sub‐subgroup	*N* from CIQUAL	*N* events^a^	% Low	% Medium	% High
	Vegetables	Vegetables raw	115	8695	0.00	99.94	0.06
		Vegetables cooked	83	7838	99.36	0.64^b^	0.00
	Fruits	Fruits rich in Vitamin C raw with peel	31	928	0.00	100.00	0.00
		Other fruits raw with peel	39	2811	0.00	100.00	0.00
Meat/Fish/Eggs	Fish and shellfish	Lean fish and shellfish raw	14	66	0.00	100.00	0.00
		Fatty fish raw	7	36	0.00	100.00	0.00
	Meat	Red meat raw	8	35	0.00	100.00	0.00
		Other meats raw	1	4	0.00	100.00	0.00
Dairy products	Milk and fresh dairy (yogurt)	Milk	16	6747	98.06	0.00	1.94^c^
		Fresh dairy	30	6729	0.00	0.00	100.00
		Fortified milk	6	344	99.71	0.00	0.29
		Fortified fresh dairy	8	722	0.00	0.00	100.00
	Cheese	Soft cheese unpasteurised	59	1688	0.00	0.00	100.00
		Soft cheese pasteurised	29	1150	0.00	98.09	1.91
		Hard cheese unpasteurised	43	2747	0.00	7.14	92.86
		Hard cheese pasteurised	20	941	0.00	96.07	3.93
		Fresh or melted cheese unpasteurised	4	81	0.00	0.00	100.00
		Fresh or melted cheese pasteurised	36	1569	90.12	9.88	0.00
		Blue cheese unpasteurised	14	361	0.00	0.00	100.00
		Blue cheese pasteurised	7	50	0.00	88.00	12.00
Mixed dishes	Mixed dishes	Mixed dishes^g^	478	11 198	95.09	4.91	0.00
Sweets and fats	Sugar sweetened foods	Sugar sweetened foods	506	32 463	99.98	0.02^d^	0.00
		Dairy dessert	18	1549	99.81	0.19^e^	0.00
	Salty and fatty foods	Salty and fatty foods	182	8668	92.42	7.33	0.25
Beverages	Sweetened drinks	Sweetened drinks	49	5377	99.50	0.50^f^	0.00
Fats and oils	Sauces	Sauces^h^	57	1577	96.89	1.46	1.65

*Note:* CIQUAL: French nutrient composition database (Agence nationale de sécurité sanitaire de l'alimentation de l'environnement et du travail (2016) Ciqual: Table de composition nutritionnelle des aliments. https://ciqual.anses.fr/).

^a^ Number of consumption events per food subgroup among children and adults.

^b^ The cooked vegetable food categorised into Medium live microbe content was sauerkraut. All other cooked vegetables were considered as Low.

^c^ The types of milk categorised into High live microbe content were curdled and fermented milk. All other milk were considered as Low.

^d^ The sugar sweetened food categorised into Medium live microbe content was a yogurt ice cream. All other sugar sweetened foods were considered as Low.

^e^ Dairy desserts categorised into Medium live microbe content were milkshakes. All other dairy desserts were categorised as Low.

^f^ The sweetened drink categorised into Medium live microbe content was smoothie based on milk and yogurt. All other sweetened drinks were considered as Low.

^g^ Mixed dishes subgroup included foods such as pizzas, hamburger and sandwiches, cantonese rice, beef stew (Boeuf bourguignon), pasta carbonara, salty cakes or tarts, etc.

^h^ Sauces subgroup included foods such as pesto, mayonnaise, curry sauce, yogurt dressing, cream sauce, salad dressing, etc.

Foods in the ANSES/CIQUAL database were assigned Low, Medium and High groups, using the classification scheme of Marco et al. (2022) and following consultations of the literature (Pertziger et al. 2025). The ANSES/CIQUAL database provides not only the food name but also a very detailed description of the preparation method. In particular, fruits and vegetables are described as fresh or cooked and/or peeled and dairy products are described as pasteurised or not. This allowed us to classify all frozen yogurt as Medium and place other yogurts in the High group. All raw fruits with peel were classified as Medium (Table 1), and peeled or cooked fruits were classified as Low (Table S1). Raw vegetables were classified as Medium, raw vegetables sprouts were classified as High and cooked vegetables (except sauerkraut) were classified as Low. Following Marco et al., unpasteurised cheeses were mainly classified as High and pasteurised cheeses were classified as Medium or Low, depending on the type of cheese. Cooked meats were all classified as ‘Low’. The classification scheme for food groups and subgroups shown in Table 1. Food groups where 100% of items had low live microbe content are listed in Table S1. Foods classified as Medium or High were also pooled together as Medium/High in the analyses. Fermented foods in the ANSES/CIQUAL database were identified as dairy products (e.g., yogurt, kefir and cheese), vegetables and legumes (e.g., tofu, miso and sauerkraut) and condiments (e.g., soya sauce, vinegar, pickle and caper).

### 2.4 Dietary Quality Scores

Dietary quality metrics were Guideline score 2 (sPNNS‐GS2 score), developed by the Programme National Nutrition Santé (Chaltiel, Adjibade, Deschamps, et al. 2019), mean adequacy ratio (MAR, %) (Bocquier et al. 2015; Vieux et al. 2013) and mean excess ratio (MER, %) (Vieux et al. 2013). The sPNNS‐GS2 score assessed compliance with the food‐based French dietary guidelines (Chaltiel, Adjibade, Deschamps, Touvier, et al. 2019). Higher sPNNS‐GS2 scores were associated with higher adherence to 2017 dietary guidelines. The MAR score for each individual was the mean of 24 nutrient adequacy ratios, each corresponding to the percentage of the French daily recommended intake (age and gender specific) for one of the 24 nutrients (i.e., α‐linolenic acid, linoleic acid, protein, EPA‐DHA, fibre, vitamin b1, b2, b3, b6, b9, b12, vitamin c, d, e, a, calcium, iodine, phosphorus, potassium, selenium, copper, iron, magnesium and zinc) (ANSES 2011, 2016b, 2021). Each nutrient adequacy ratio was truncated at 100 so that a high intake of one nutrient could not compensate for a low intake of another. MAR ranged from 0 (no nutrient intake) to 100 (coverage of all 24 recommended intakes). The MER was the mean percentage of excess in sodium, saturated fatty acids (SFA) (ANSES 2011, 2021) and free sugars (World Health Organization 2015). The recommendation was expressed on a daily basis for sodium, and in percentage of total energy for free sugars and saturated fats.

### 2.5 Statistical Analyses

INCA3 participants were assigned into three groups based on their consumption of live microbe foods: (1) Consumers of Medium foods; (2) Consumers of High foods; and (3) Consumers of Medium or High foods or both (referred to as ‘Medium/High’ throughout). Alcoholic beverages were excluded from the analyses.

Mean intakes (g/day) of Medium, High and Medium/High foods by children and adults were compared by socio‐demographic variables, using generalised linear models (GLM). To identify which foods contributed the most to Medium/High food consumption, mean intakes (in g/d) of Medium, High and Medium/High foods were estimated by food groups and subgroups as well in percentage of total intake of Medium, High and Medium/High foods. The contribution (in percentage) of Medium, High and Medium/High foods to total nutrient intakes was estimated for adults and children.

Food consumption and nutrient intakes were assessed by level of Medium/High food consumption. Individuals were categorised into tertiles based on their dietary intake in g/d of Medium/High food consumption. Then, both among adults and children, average food group and subgroup intakes (in g/d) were estimated by tertile, as well as dietary quality scores.

Differences in intakes of foods with live microbes were assessed by general linear models, without adjustment and adjusted for total energy intakes, Socio‐professional category (SCP), Income per consumption unit (CU) and BMI. Adjusted mean values and distributions (25th, 50th and 75th percentiles) of intakes of fermented foods were estimated among children and adults. Nutritional quality of the diet was assessed with the three dietary quality scores and also using percentages of individuals meeting French nutritional recommended values by tertiles of intake of Medium/High foods. All analyses accounted for the complex survey design of INCA3 data and were representative of the French metropolitan population. Data analyses used R software version 4.4.

## 3 Results

### 3.1 INCA 3 Participant Characteristics

Table 2 shows socio‐demographic characteristics of the INCA3 sample for children and adults. The sample was composed of 1775 children (aged ≥ 4 years) and 2121 adults (aged 18–79 years). The socio‐demographic variables of interest were sex, age (for adults), SPC, income per CU and BMI. This was a representative sample of the French population in 2014–2015. Percentages were weighted according to sampling weights in the INCA3 survey.

**TABLE 2:** Socio‐demographic and economic characteristics of the INCA 3 sample.

Characteristic		Children (< 18 years)		Adults (> 18 years)	
		*n*	%	*n*	%
All		1775		2121	
Sex	Male	903	51.2	887	48.5
	Female	872	48.8	1234	51.5
Age	18–44 years	—	—	783	45.9
	45–64 years	—	—	827	36.5
	65–79 years	—	—	511	17.6
Socio‐professional category^a^	Low	654	40.6	499	27.6
	Middle	522	26.1	435	22.1
	High	433	21.3	349	15.0
	Inactive	166	12.2	838	35.3
Income (Euros/month)^a^	< 900	393	27.5	358	23.1
	900–1340	452	26.2	421	21.4
	1340–1850	499	25.9	471	21.8
	> 1850	300	13.0	708	26.9
	Missing	131	7.4	163	6.8
BMI (kg/m^2^)^b^	< 18	190	11.4	53	2.9
	18–25	1277	71.1	993	44.8
	25–30	239	13.6	718	35.0
	> 30	69	3.9	352	17.1
	Missing	—	—	5	0.2

*Note:* INCA3: French Individual and National Food Consumption Survey 3 (ANSES (2019) Données de consommations et habitudes alimentaires de l'étude INCA 3. Available at: https://www.data.gouv.fr/datasets/donnees‐de‐consommations‐et‐habitudes‐alimentaires‐de‐letude‐inca‐3/ (accessed 12 December 2025)).

^a^ Socio‐professional category and income level of the household referent.

^b^ For children BMI classes refers to thinness; normal; overweight and obesity, respectively, according to the international definition established by the Childhood Obesity Working Group of the International Obesity Task Force and specific to age and sex.

### 3.2 Consumption of Medium and Medium/High Food Groups

Over the 3 days of data collection period 98.4% of adults and 97.7% of children consumed foods classed as having Medium/High content of live microbes. Consumption prevalence for Medium foods was 91.7% of adults and 81.1% of children. Consumption prevalence for High foods (mostly fermented dairy products) was 88.2% for adults and 88.5% for children.

**FIGURE 1:**
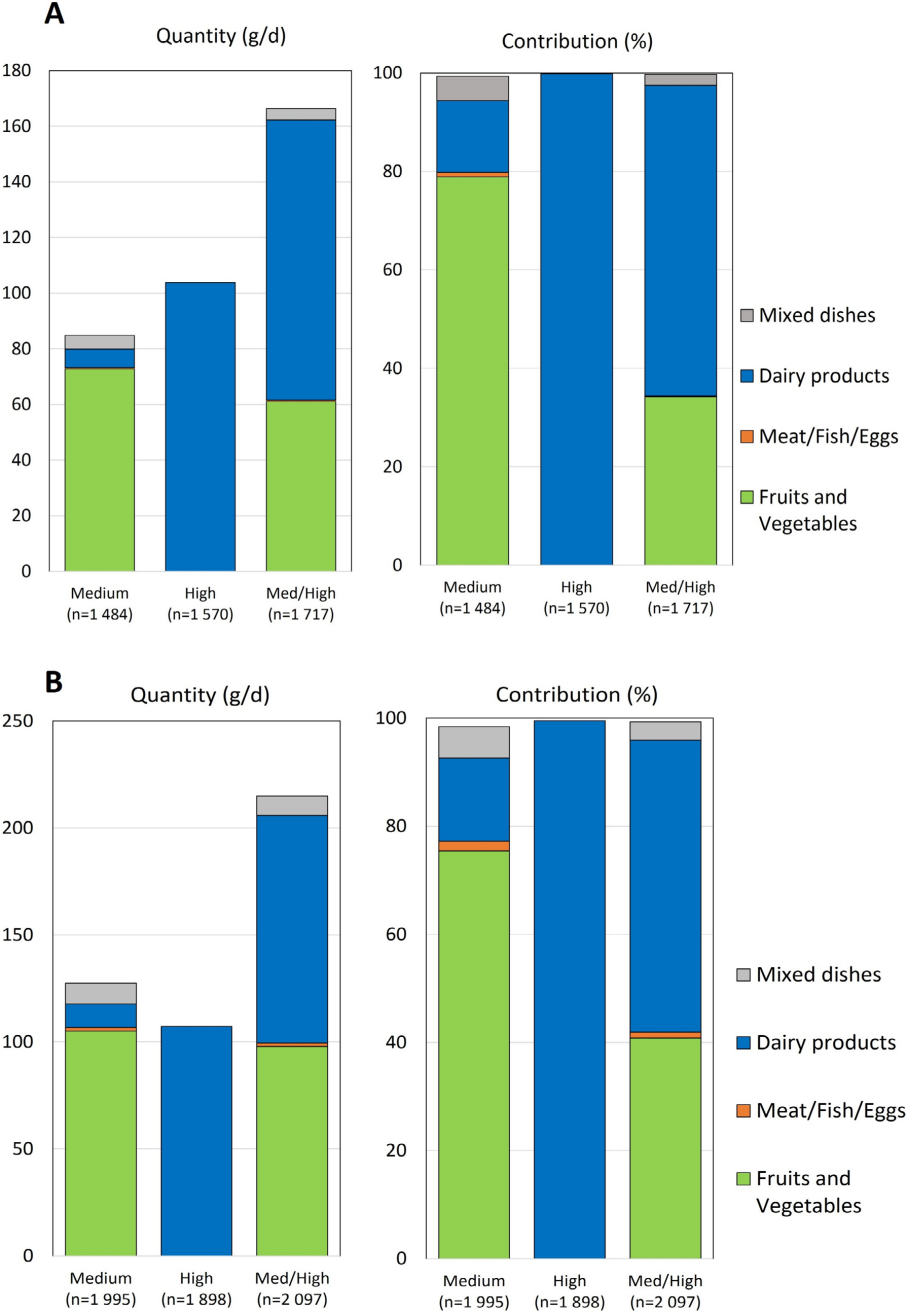
Mean consumption among children (A) and adult (B) consumers of Medium, High, and Medium/High foods by food groups in g per day and as percentage contribution to total intakes of Medium, High and Medium/High foods.

Total consumption of Medium/High foods was 166.8 g/day for children and 215.9 g/day for adults. Figure 1 shows amounts consumed (in g/day) and the relative contribution of food groups (in %). Among children, fruits and vegetables contributed 73 g/day to the total intake of Medium foods (85 g/day), followed by dairy products (6.7 g/day) and mixed dishes (4.9 g/day). Dairy products were by far the leading contributor to the High foods class (100 g/day), followed by vegetables (61.1 g/day). Among adults, fruits and vegetables contributed 105.1 g/day to the total intake of Medium foods (129 g/day), followed by dairy products (11.0 g/day) and mixed dishes (9.7 g/day). Dairy products were by far the leading contributor to the High foods class (107.2 g/day), followed by vegetables (97.9 g/day). Data shown in Figure 1 are contained in Tables S2 and S3.

### 3.3 Consumption of Medium and High Foods by Age Group

**FIGURE 2:**
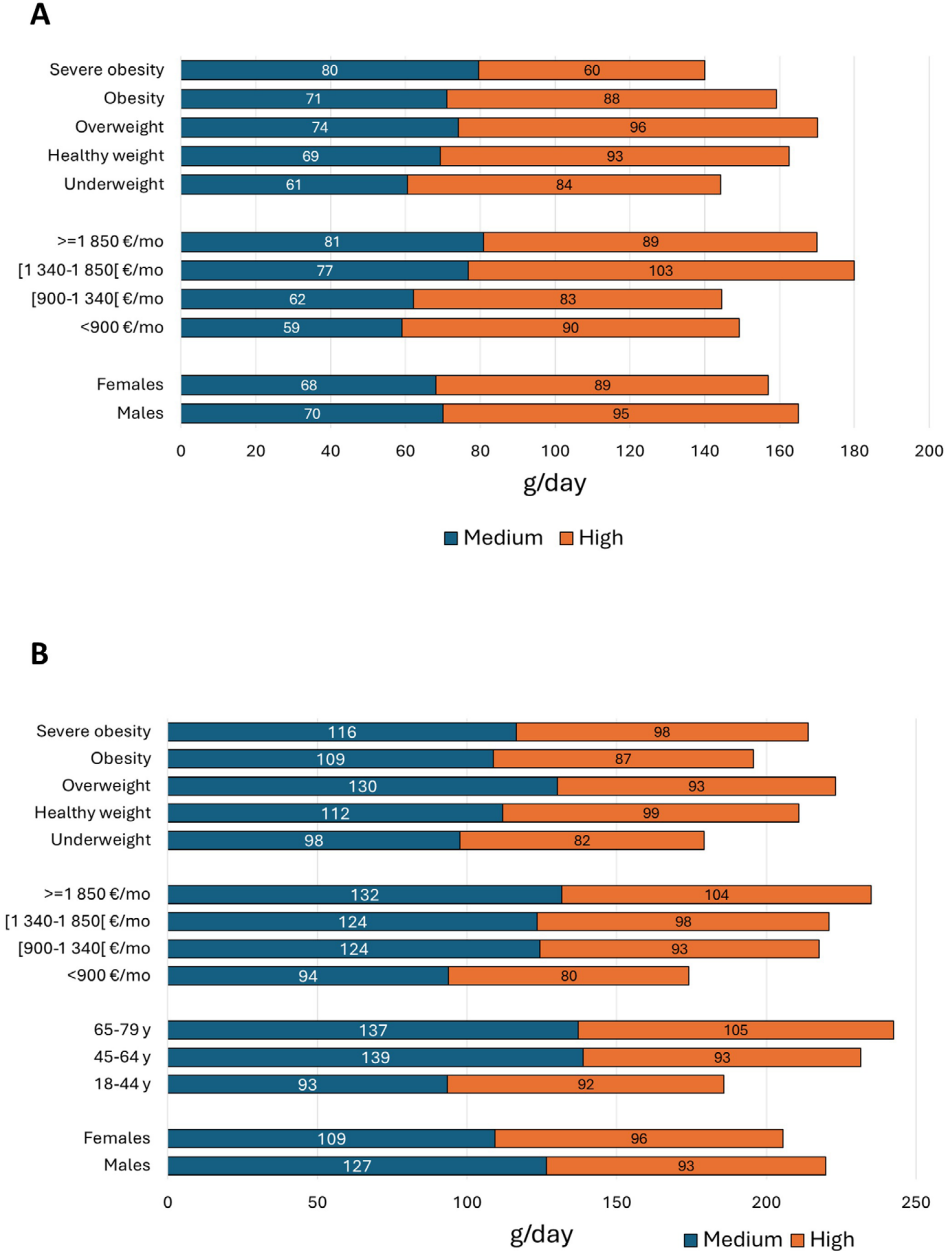
Mean consumption (g/d) of Medium and High foods by sociodemographic variables among children (A) and adults (B).

Mean amounts consumed (in g/day) of Medium and High foods by age group are shown in Figure 2. There was a significant effect of age for both Medium and High foods, with significantly highest amounts observed for older adults. Higher consumption of Medium/High foods was associated with higher socioeconomic status and higher incomes. No differences were observed by sex and BMI (Data are in Table S4).

### 3.4 Contribution of Medium/High Foods to Nutrient Intakes

Medium/High foods contributed 8.5% (159.9 kcal/d) of dietary energy among children and 10.7% (223.7 kcal/d) of dietary energy among adults. There was little variation by demographics (Tables S5 and S6). Medium foods contributed 2.7% of dietary energy among children and 4.2% among adults. High foods contributed 5.9% of dietary energy among children and 6.5% among adults.

**FIGURE 3:**
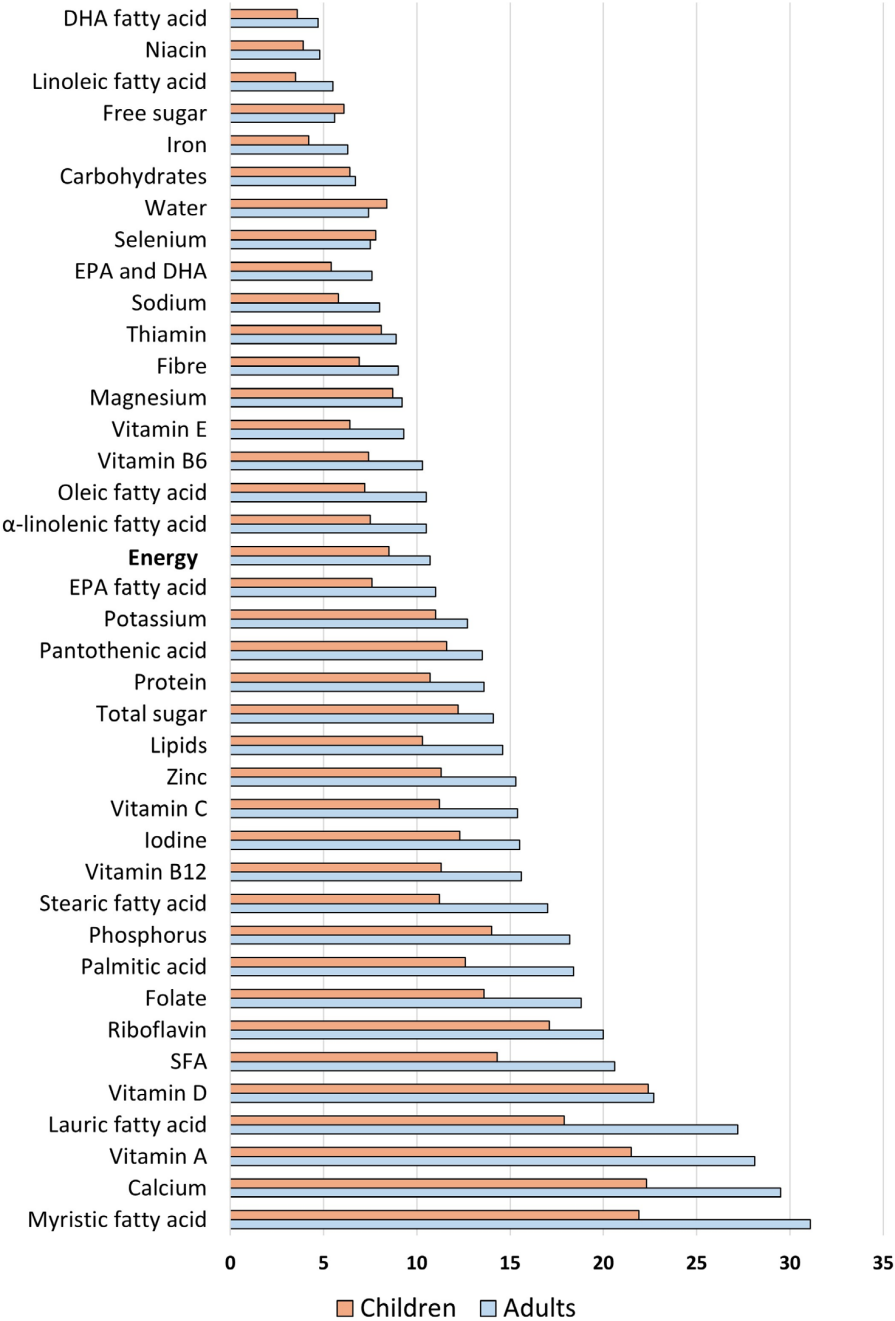
Percent contribution of Medium/High foods to total energy and total intakes of that nutrient or micronutrient among children and adults. Nutrients are sorted by ascending order of contribution to adult diets.

Figure 3 shows that Medium/High foods contributed more than 20% to the total daily intake of myristic fatty acid, calcium, vitamin A, lauric fatty acid, vitamin D, saturated fatty acids and riboflavin intakes among adults, and to vitamin D, calcium, myristic fatty acid and vitamin A among children (Figure 3, Table S6).

### 3.5 Dietary Patterns by Tertiles of Consumption of Medium/High Foods

**FIGURE 4:**
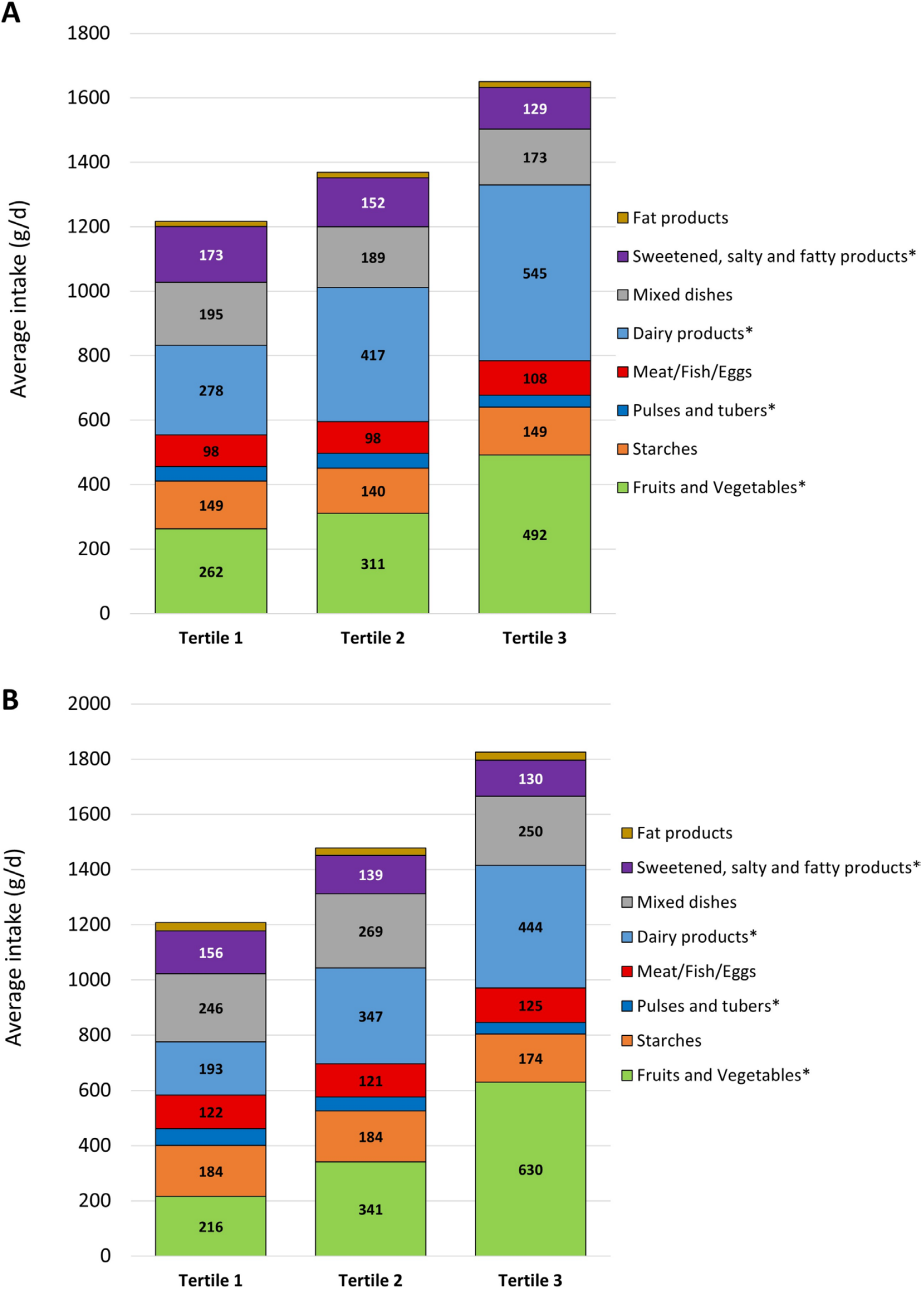
Mean intakes (g/d) of food groups (without beverages) by tertiles of Medium/High foods consumption among children (A) and adults (B). Values are mean adjusted on total energy intakes, ICU, SPC and BMI. *Asterisks denote significant differences between tertiles ICU, income per consumption unit; SPC, socio‐professional category (SPC).

For children, tertile cut offs (in g/day) for Medium/High foods were T1 (0–98 g); T2 (98–191 g/day) and T3 (> 191 g/day). For adults, tertile cut offs were T1 (0–133 g/day); T2 (133–248 g/day) and T3 (> 248 g/day). Consumption data were adjusted for energy intake and socio‐demographics. In addition to fruits, vegetables and dairy, children and adults in the highest tertile consumed less mixed dishes and products identified as sweetened, salty and fatty (including dairy desserts, sweet products such as biscuits/cakes or savoury snack biscuits) (Figure 4, Table S7). Adults in the highest tertile consumed more whole grain cereals, fish and crustaceans, whereas children consumed more pulses. Both adults and children in the highest tertile consumed less sweetened drinks.

### 3.6 Dietary Quality by Levels of Medium/High Foods Consumption

**FIGURE 5:**
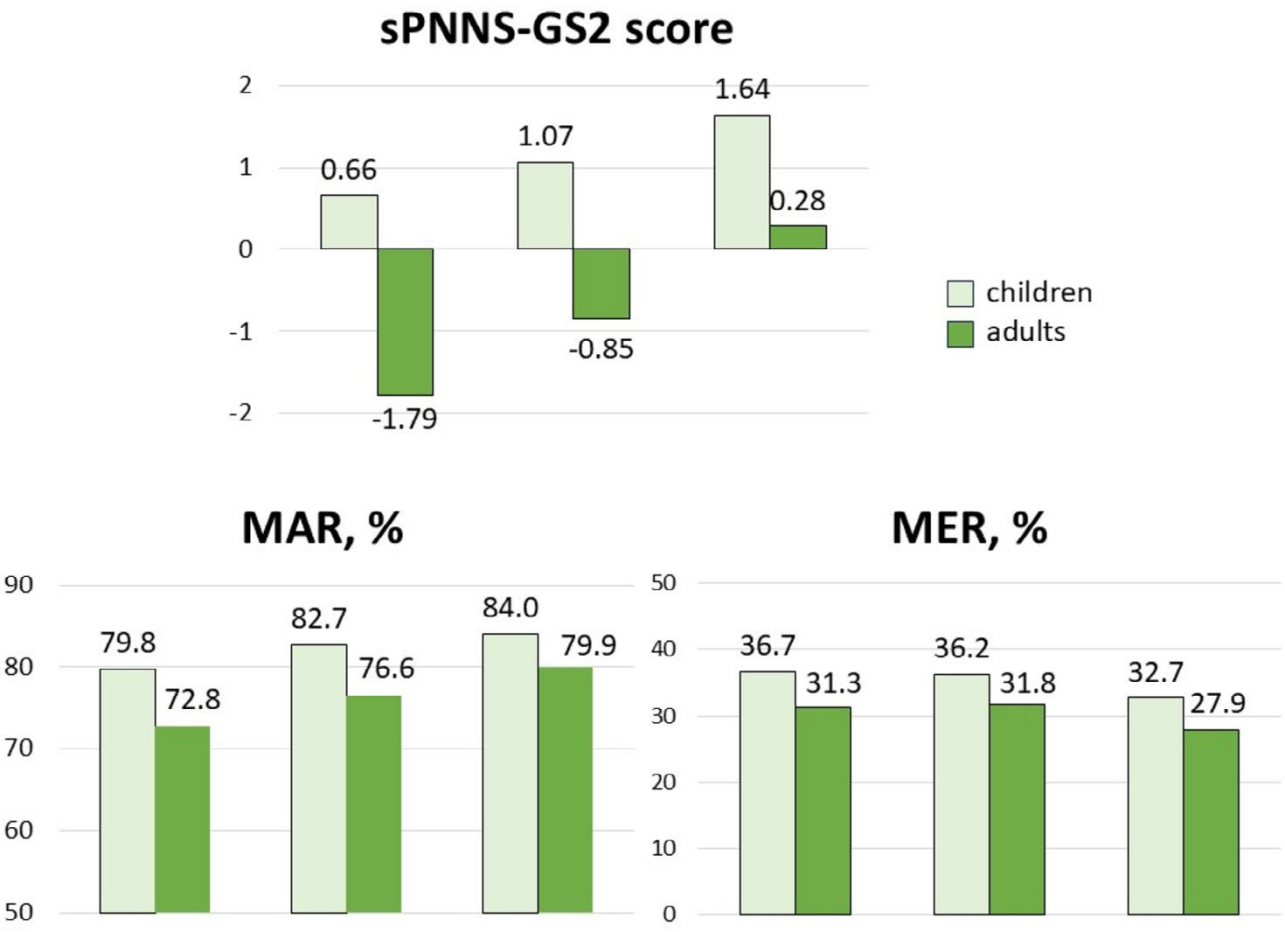
Average sPNNS‐GS2 scores^$^, MAR^§^ and MER^μ^ by tertile of Medium/High foods consumption among children and adults. ^$^Guideline score 2 (sPNNS‐GS2 score), developed by the Programme National Nutrition Santé, assessed compliance with the 2017 food‐based French dietary guidelines. Higher sPNNS‐GS2 scores were associated with higher adherence. ^§^Mean Adequacy Ratio (MAR) score is the mean adequacy ratios to daily recommendations for 24 nutrients. It ranges from 0% to 100% (i.e., a score of 100% means that all the recommendations for 24 nutrients are reached) ^μ^Mean Excess Ratio (MER) score estimated the mean excess in sodium, free sugar and saturated fats. MER score is strictly positive. A high value indicates a high amount of nutrients to limit in the diet. A score of 0% means that the diet does not exceed the maximal recommended values for the three nutrients.

For both children and adults, dietary quality indicators were significantly better among higher consumers of Medium/High foods, with a higher MAR, sPNNS‐GS2 and a lower MER (*p* value < 0.001 for sPNNS scores and MAR (children and adults); *p* value = 0.018 (children) and *p* value = 0.0016 (adults) for MER). Means were adjusted for energy intakes, socio‐professional category, income per consumption unit and BMI (Figure 5, Table S8).

Higher consumption of Medium/High foods was associated with higher compliance with recommended nutrient values (Table S9). Individuals who consumed more Medium/High foods (T3) had more nutrient‐rich diets. Regarding nutrients to limit, the trend was not significant for most of the nutrients. For sodium, higher consumers of Medium/High foods had a lower percentage of individuals not exceeding the recommended value.

### 3.7 Consumption of Fermented Products of Interest

Yogurt was consumed by 79% of children, with a mean intake of 73.2 g/day, while 68.6% of adults consumed yogurt, averaging 65.5 g/day (Table 3). Cheese was consumed by 60.5% of children, who ate a mean of 47.8 g/day, and was consumed by 76.3% of adults, with a mean intake of 94.1 g daily.

**TABLE 3:** Distribution of the consumption of fermented products of interest (grams/day), and percentage of consumers among children and adults.

	Fermented products	%^a^	Mean^a^	Std	95% LCL^b^	95% UCL^c^	Min	P5	P25th	Median	P75th	P95	Max
Children (*n* = 1775)	Yogurt	79.0	73.2	67.4	68.5	77.9	0.0	0.0	22.1	64.4	105	200	441
	Cheese	60.5	47.8	66.7	43.1	52.5	0.0	0.0	0.0	23.1	71.9	187	486
	Kefir	0.0	0.0	0.0	0.0	0.0	0.0	0.0	0.0	0.0	0.0	0.0	0.0
	Tofu, miso, sauerkraut	0.4	0.1	1.0	0.0	0.1	0.0	0.0	0.0	0.0	0.0	0.0	27.4
	Condiments	10.1	0.3	1.2	0.2	0.3	0.0	0.0	0.0	0.0	0.0	1.5	20.6
	Not fermented	100	1712	571	1659	1764	476	1007	1332	1629	1977	2715	4939
Adults (*n* = 2121)	Yogurt	68.6	65.5	68.3	60.7	70.3	0	0	0	52.7	104	199	567
	Cheese	76.3	94.1	101	88.2	100	0	0	11	69.2	140	298	711
	Kefir	0.1	0.1	5.0	0	0.3	0	0	0	0	0	0	354
	Tofu, miso, sauerkraut	1.9	0.4	3.6	0.2	0.5	0	0	0	0	0	0	96.6
	Condiments	21.0	0.7	2.3	0.6	0.9	0	0	0	0	0	5.6	31.9
	Not fermented	100	1848	682	1800	1896	269	908	1339	1760	2261	3007	5575

*Note:* INCA3: French Individual and National Food Consumption Survey 3 (ANSES (2019) Données de consommations et habitudes alimentaires de l'étude INCA 3. Available at: https://www.data.gouv.fr/datasets/donnees‐de‐consommations‐et‐habitudes‐alimentaires‐de‐letude‐inca‐3/ (accessed 12 December 2025)).

^a^ Percentages of consumers and mean intake were weighted according to sampling weights in the INCA3 survey.

^b^ Lower confidence limit.

^c^ Upper confidence limit.

Tofu, miso and sauerkraut were rarely consumed, with only 0.4% of children and 1.9% of adults reporting intake, averaging 0.1 g/day and 0.4 g/day, respectively. Condiments were consumed by 10.1% of children, with a mean consumption of 0.3 g/day, and by 21% of adults, with a mean consumption of 0.7 g/day. Kefir was not consumed by any children and was rarely consumed by adults, with only 0.1% reporting intake and a mean of 0.1 g/day.

## 4 Discussion

The present exposure to foods with live microbes needs to be compared to previous data. In the present study, Medium/High foods were consumed by 97.7% of children and 98.4% of adults over 3 days. Mean per capita intake of Medium/High foods was 166.8 g/day for children and 215.9 g/day for adults. Mean per capita intake of High foods (i.e., yogurt and unpasteurised cheese) was approximately 100 g/day. Medium/High foods contributed > 5% of daily energy but substantial amounts of calcium, vitamin A and vitamin D.

Marco et al. (2022) found that 59% of children and 67% of adults consumed Medium/High foods on 1 day of NHANES. Mean per capita intake of Medium/High foods was 85 g/day for children and 127 g/day for adults. Mean per capita intake of High foods (i.e., dairy products) was only 16 g/day for children and 21 g/day for adults. Medium/High foods contributed less than 5% of energy and > 10% of some key nutrients. A more recent study of Swiss adults (Pertziger et al. 2025) placed mean intake of Medium/High foods at 269.3 g/d. Medium/High foods contributed 12.3% of energy and > 20% of daily intake of β‐carotene, vitamins A, C, B12, folate, calcium and saturated fat.

There are two preliminary conclusions. First, the consumption of foods with live microbes in France was much greater than in the US. Second, whereas live microbes in the US diet came mostly from fresh produce, live microbes in the French diet came mostly from yogurt and unpasteurised cheese.

The present study used the procedures of Marco et al. (2022) to assign foods into Low, Medium and High groups. Using the US Department of Agriculture Food and Nutrient Database for Dietary Studies (FNDDS), Marco et al. (2022) estimated the amounts of viable microbes expected to be present in pasteurised foods (< 10 4 CFU/g), fresh fruits and vegetables eaten unpeeled (104–107 CFU/g) and unpasteurised fermented foods and probiotic supplements (> 107 CFU/g). Based on those criteria, Marco et al. (2022) assigned 8954 food codes out of 9388 (95%) to the Low group. Those were processed foods, hot milk, cooked meat and seafood dishes; uncooked mixed salads, and peeled fruits and vegetables. The Medium group was composed of fresh vegetables (41%), fruits (39%) and beverages, condiments and sauces (> 10%), along with some fermented foods (miso and sauerkraut). The High group included yogurt, sour cream and most cheeses except for pizza‐type cheeses and pasteurised American (processed) cheese. Foods with cheese as an ingredient were classified as either Low or Medium, depending on the amount of cheese in the food product.

The ANSES/CIQUAL database differs from FNDDS in listing more dairy products, and a much greater variety of unpasteurized cheeses. It also provides more detail on the mode of preparation, allowing for finer distinctions among categories. Accordingly, 2364 foods out of 2812 (84%) were assigned to the Low microbe group. Vegetables and fruit were the top sources of Medium codes (75%). As expected, yogurt and unpasteurised cheeses were the top sources of High codes (99%).

By contrast, the consumption of such fermented foods as tofu, miso, sauerkraut, pickles and other condiments was very low.

Consistent with Marco et al. (2022) and Pertziger et al. (2025), Medium/High foods contributed more nutrients than calories to the total diet. This food group, composed primarily of vegetables and fruit (Medium) and yogurt and unpasteurised cheese (High) can therefore be described as inherently nutrient rich. Not surprisingly, higher consumption of nutrient rich foods was associated with higher quality diets.

One metric of diet quality, specifically designed to assess compliance with the French National Plan for Nutrition and Health (Plan National Nutrition Santé or PNNS) (Delamaire et al. 2019) showed that diets higher in Medium/High foods were higher in nutrients to encourage, but also higher in sodium. Previous studies have not formally linked the consumption of foods with live microbes (dairy, vegetables and fruit) to diet quality metrics. However, such a result would be expected given the contribution of yogurt, cheese and fresh produce to high quality diets.

The present analysis had limitations. Both NHANES and INCA 3 dietary surveys rely on 24 h dietary recalls, that is to say self‐report. For younger children, the self‐report is provided by proxy. Both studies are cross sectional, not allowing for the drawing of any causal relations between diets and health. The categorisation of foods by putative microbe content was, of necessity, simplistic. Both studies used a simplified approach to group foods into three classes; the assignment having been based on previous studies. However, it is important to note that the variability in live microbe content across different foods is substantial. Fermented foods such as yogurt, kefir, sauerkraut and kimchi exhibit concentrations depending on the fermentation process, strain specificity and post‐production handling (Marco et al. 2022). Similarly, raw fruits and vegetables show significant fluctuations in live microbe levels, influenced by soil microbiota, farming practices (e.g., organic vs. conventional) and post‐harvest conditions (Delitte et al. 2021; Leff and Fierer 2013). We have adapted a model that was devised for the US to the corresponding nutrient composition data in France. The intake of live microbes may be very different depending on agricultural practices, food transportation and storage.

It is also worth mentioning that the US Food and Drug Administration (FDA) does not at this time recognise probiotics or fermented foods as a distinct regulatory category. In 2024, the FDA allowed a qualified health claim for yogurt in relation to reduced risk of type 2 diabetes, but only with explicit disclaimer about limited evidence (U.S. Food and Drug Administration 2024).

## 5 Conclusion

The consumption of foods likely to contain live microbes (dairy, vegetables and fruit) was substantially higher in France compared to the US. This was due to much higher consumption in France of fermented dairy products, notably yogurt and cheese. Medium/High foods provided < 5% of total daily energy but > 10% of some key micronutrients. Consumption of other fermented foods was low. Medium/High foods, notably yogurt, cheese, vegetables and fruit can be viewed as markers of healthier diet and their consumption is associated with improved diet quality metrics.

## Author Contributions

A.D. and M.M. conceptualised the study. R.G. and R.P. analysed the dataset. A.D. acted as lead writer of the manuscript. M.M. and F.V. interpret results. All authors contributed to the manuscript preparation and have read and agreed to the published version of the manuscript.

## Funding

This work was supported by The Kraft Heinz Company and views expressed in this manuscript are those of the authors and do not necessarily reflect the position or policy of The Kraft Heinz Company. We thank Kayla Hooker for technical support.

## Conflicts of Interest

R.G., M.M., F.V. and R.P. are with MS‐Nutrition, a startup that provides custom analyses of research data that is located in Marseille, France. A.D. is the developer of the Naturally Nutrient‐Rich (NNR) and the Nutrient‐Rich Food (NRF) nutrient profiling models and is or has been a member of scientific advisory panels for BEL, Lesaffre, Nestlé, FrieslandCampina Institute, National Pork Board and Carbohydrate Quality Panel supported by Potatoes USA. A.D. has worked with Ajinomoto, Ayanabio, FoodMinds, KraftHeinz, Lesaffre, Meiji, MS‐Nutrition, Nutrition Impact LLC, Nutrition Institute, PepsiCo, Samsung and Soremantec on quantitative ways to assess nutrient density of foods. The funders had no role in the design of the study; in the collection, analyses or interpretation of data; in the writing of the manuscript; or in the decision to publish the results.

## Supporting information


**Table S1:** Food sub‐subgroups with exclusively low live microbe content. Food items from the CIQUAL nutrient compositon database.
**Table S2:** Mean consumption (in grams per day) of medium, high and medium/high foods by food groups and selected categories (mean value > 0.2 g).
**Table S3:** Mean contribution (%) to total medium, high and medium/high consumption by selected food groups (mean value > 0.5%).
**Table S4:** Mean consumption (grams per day) of medium, high and medium/high foods by sociodemographic variables.
**Table S5:** Mean total consumption (in kcal per day) and mean consumption from medium/high foods by sociodemographic variables for children (*n* = 1775) and adults (*n* = 2121).
**Table S6:** Contribution (%) of medium, high, medium/high foods to total nutrient intakes.
**Table S7:** Mean intakes (g/day) by food groups and subgroups and tertile of medium/high consumption among adults, and adjusted means (on total energy intakes, IUC, PCS and BMI).
**Table S8:** Mean scores by tertile of medium/high consumption among adults and adjusted means (on total energy intakes—except for energy IUC PCS and BMI).
**Table S9:** Percentage of adults and children meeting the minimal recommended (RV) value by tertile^$^ of medium/high food consumption.

## Data Availability

The data that support the findings of this study are openly available from Agence nationale de sécurité sanitaire de l'alimentation de l'environnement et du travail (ANSES) at: https://www.anses.fr/system/files/NUT2006sa0359Ra.pdf, ANSES (2011); https://ciqual.anses.fr, ANSES (2016a); https://www.anses.fr/en/system/files/NUT2012SA0103Ra‐2.pdf, ANSES (2016b); https://www.data.gouv.fr/datasets/donnees‐de‐consommations‐et‐habitudes‐alimentaires‐de‐letude‐inca‐3/, ANSES (2019); https://www.anses.fr/fr/system/files/NUT2018SA0238Ra.pdf, ANSES (2021).
