# Supplementary material for: Association Between Dietary Live Microbes and Diet Quality Among Children and Adults in France
Source: Nutr Bull. 2026 May 23;51(2):245–57. doi: 10.1111/nbu.70057 (PMC13254697; doi:10.1111/nbu.70057)
Supplement: Supplementary file 1 — Table S1: Food sub‐subgroups with exclusively low live microbe content. Food items from the CIQUAL nutrient compositon database. Table S2: Mean consumption (in grams per day) of medium, high and medium/high foods by food groups and selected categories (mean value > 0.2 g). Table S3: Mean contribution (%) to total medium, high and medium/high consumption by selected food groups (mean value > 0.5%). Table S4: Mean consumption (grams per day) of medium, high and medium/high foods by sociodemographic variables. Table S5: Mean total consumption (in kcal per day) and mean consumption from medium/high foods by sociodemographic variables for children (n = 1775) and adults (n = 2121). Table S6: Contribution (%) of medium, high, medium/high foods to total nutrient intakes. Table S7: Mean intakes (g/day) by food groups and subgroups and tertile of medium/high consumption among adults, and adjusted means (on total energy intakes, IUC, PCS and BMI). Table S8: Mean scores by tertile of medium/high consumption among adults and adjusted means (on total energy intakes—except for energy IUC PCS and BMI). Table S9: Percentage of adults and children meeting the minimal recommended (RV) value by tertile$ of medium/high food consumption. [file NBU-51-245-s001.docx]

Supplemental Table 1 Food sub-subgroups with exclusively low live microbe content. Food items from the CIQUAL nutrient compositon database.

| **Food groups** | **Food subgroup** | **Food sub-subgroup** | **N items** | **N events** | **% Low** | **% Medium** | **% High** |
| --- | --- | --- | --- | --- | --- | --- | --- |
| Fruits and vegetables | Fruits | Fruits rich in Vitamin C raw without peel | 16 | 3410 | 100.00 | 0.00 | 0.00 |
|  |  | Fruits rich in Vitamin C cooked | 13 | 111 | 100.00 | 0.00 | 0.00 |
|  |  | Other fruits raw without peel | 41 | 3234 | 100.00 | 0.00 | 0.00 |
|  |  | Other fruits cooked | 32 | 2384 | 100.00 | 0.00 | 0.00 |
|  |  | Dry fruits | 11 | 205 | 100.00 | 0.00 | 0.00 |
|  | Nuts | Nuts | 30 | 866 | 100.00 | 0.00 | 0.00 |
|  | Fruit juices | Fruit juices | 4 | 5398 | 100.00 | 0.00 | 0.00 |
| Starches | Whole grains | Whole grain bread | 35 | 3308 | 100.00 | 0.00 | 0.00 |
|  |  | Whole grain products (pasta, rice, semolina) | 13 | 201 | 100.00 | 0.00 | 0.00 |
|  | Refined grains | Refined bread | 61 | 14178 | 100.00 | 0.00 | 0.00 |
|  |  | Refined products (pasta, rice, semolina) | 67 | 5983 | 100.00 | 0.00 | 0.00 |
| Legumes and tubers | Potatoes and tubers | Potatoes and tubers | 15 | 3399 | 100.00 | 0.00 | 0.00 |
|  | Legumes | Legumes | 16 | 598 | 100.00 | 0.00 | 0.00 |
| Meat/Fish/Eggs | Fish and shellfish | Lean fish and shellfish cooked | 84 | 1882 | 100.00 | 0.00 | 0.00 |
|  |  | Fatty fish cooked | 25 | 963 | 100.00 | 0.00 | 0.00 |
|  | Meat | Red meat cooked | 94 | 4211 | 100.00 | 0.00 | 0.00 |
|  |  | Other meats cooked | 82 | 2881 | 100.00 | 0.00 | 0.00 |
|  |  | Offal | 16 | 223 | 100.00 | 0.00 | 0.00 |
|  |  | Cured meat | 85 | 5548 | 100.00 | 0.00 | 0.00 |
| Sweets and fats | Sugar sweetened foods | Fortified sugar sweetened foods | 44 | 2289 | 100.00 | 0.00 | 0.00 |
|  | Salty and fat foods | Salt | 11 | 3834 | 100.00 | 0.00 | 0.00 |
| Beverages | Water | Tap water | 4 | 21841 | 100.00 | 0.00 | 0.00 |
|  |  | Bottled water | 19 | 15638 | 100.00 | 0.00 | 0.00 |
|  | Hot drinks | Hot drinks | 35 | 14086 | 100.00 | 0.00 | 0.00 |
| Fats and oils | Vegetable fats | Olive oil | 1 | 2721 | 100.00 | 0.00 | 0.00 |
|  |  | Rapeseed-flax-walnut-oils | 4 | 537 | 100.00 | 0.00 | 0.00 |
|  |  | Other oils | 16 | 948 | 100.00 | 0.00 | 0.00 |
|  |  | Other vegetable fats | 13 | 1291 | 100.00 | 0.00 | 0.00 |
|  |  | Other fortified vegetable fats | 5 | 936 | 100.00 | 0.00 | 0.00 |
|  |  | Salad dressings | 18 | 3641 | 100.00 | 0.00 | 0.00 |
|  | Animal fats | Animal fats | 18 | 6489 | 100.00 | 0.00 | 0.00 |
| Alternative proteins | Alternative proteins | Alternative proteins | 24 | 378 | 100.00 | 0.00 | 0.00 |
|  |  |  |  |  |  |  |  |

Supplemental table 2. Mean consumption (in grams per day) of Medium, High and Medium/High foods by food groups and selected categories (mean value > 0.2 g)

| **Children (n=1775)** | | | | | | | | | | | | |
| --- | --- | --- | --- | --- | --- | --- | --- | --- | --- | --- | --- | --- |
| **Food groups and categories** | **Medium** | | | | **High** | | | | **Medium/High** | | | |
|  | **Mean** | **Std** | **95%LCL*** | **95%UCL*** | **Mean** | **Std** | **95%LCL** | **95%UCL** | **Mean** | **Std** | **95%LCL** | **95%UCL** |
| Fruits and vegetables | 72.8 | 73.9 | 68.2 | 77.4 | 0.0 | 0.0 | 0.0 | 0.0 | 61.1 | 72.8 | 56.6 | 65.7 |
| Vegetables | 33.0 | 39.8 | 30.2 | 35.8 | 0.0 | 0.0 | 0.0 | 0.0 | 27.7 | 38.5 | 25.1 | 30.3 |
| Fruits | 39.8 | 57.0 | 36.2 | 43.4 | 0.0 | 0.0 | 0.0 | 0.0 | 33.4 | 54.3 | 30.1 | 36.7 |
| Fish/Meat/Eggs | 0.4 | 3.4 | 0.2 | 0.7 | 0.0 | 0.0 | 0.0 | 0.0 | 0.4 | 3.1 | 0.1 | 0.6 |
| Fish and shellfish | 0.3 | 2.9 | 0.1 | 0.5 | 0.0 | 0.0 | 0.0 | 0.0 | 0.3 | 2.7 | 0.1 | 0.4 |
| Meat | 0.1 | 1.7 | 0.0 | 0.2 | 0.0 | 0.0 | 0.0 | 0.0 | 0.1 | 1.6 | 0.0 | 0.2 |
| Dairy products | 6.7 | 13.4 | 5.6 | 7.9 | 103.8 | 74.0 | 98.8 | 108.8 | 100.7 | 77.9 | 95.1 | 106.2 |
| Milk and fresh dairy | 0.0 | 0.0 | 0.0 | 0.0 | 94.2 | 74.0 | 89.2 | 99.2 | 86.2 | 75.5 | 81.0 | 91.4 |
| Cheese | 6.7 | 13.4 | 5.6 | 7.9 | 9.6 | 14.4 | 8.6 | 10.6 | 14.4 | 19.9 | 13.0 | 15.9 |
| Mixed dishes | 4.9 | 20.3 | 3.6 | 6.3 | 0.0 | 0.0 | 0.0 | 0.0 | 4.1 | 18.7 | 3.0 | 5.3 |
| **Adults (n=2121)** | | | | | | | | | | | | |
| **Food groups and categories** | **Medium** | | | | **High** | | | | **Medium/High** | | | |
|  | **Mean** | **Std** | **95%LCL** | **95%UCL** | **Mean** | **Std** | **95%LCL** | **95%UCL** | **Mean** | **Std** | **95%LCL** | **95%UCL** |
| Fruits and vegetables | 105.1 | 112.5 | 99.1 | 111.1 | 0.0 | 0.4 | -0.0 | 0.0 | 97.9 | 111.8 | 91.3 | 104.5 |
| Vegetables | 58.2 | 65.6 | 54.8 | 61.5 | 0.0 | 0.4 | -0.0 | 0.0 | 54.2 | 65.0 | 50.9 | 57.6 |
| Fruits | 46.9 | 80.4 | 41.9 | 52.0 | 0.0 | 0.0 | 0.0 | 0.0 | 43.7 | 78.5 | 38.6 | 48.9 |
| Fish/Meat/Eggs | 1.7 | 9.2 | 1.1 | 2.3 | 0.0 | 0.0 | 0.0 | 0.0 | 1.6 | 8.9 | 1.0 | 2.2 |
| Fish and shellfish | 1.2 | 7.8 | 0.6 | 1.9 | 0.0 | 0.0 | 0.0 | 0.0 | 1.2 | 7.6 | 0.6 | 1.7 |
| Meat | 0.4 | 4.9 | 0.2 | 0.7 | 0.0 | 0.0 | 0.0 | 0.0 | 0.4 | 4.7 | 0.2 | 0.6 |
| Dairy products | 11.0 | 17.6 | 9.9 | 12.1 | 107.2 | 78.0 | 101.0 | 113.5 | 106.4 | 82.0 | 100.2 | 112.5 |
| Milk & fresh dairy | 0.0 | 0.0 | 0.0 | 0.0 | 86.9 | 78.2 | 80.9 | 93.0 | 78.0 | 78.7 | 72.3 | 83.6 |
| Cheese | 11.0 | 17.6 | 9.9 | 12.1 | 20.3 | 24.6 | 18.6 | 22.0 | 28.4 | 29.8 | 26.6 | 30.2 |
| Mixed dishes | 9.7 | 36.7 | 7.3 | 12.1 | 0.0 | 0.0 | 0.0 | 0.0 | 9.1 | 35.5 | 6.8 | 11.3 |

- LCL Lower confidence interval; UCL Upper confidence interval

Supplemental Table 3. Mean contribution (%) to total Medium, High and Medium/High consumption by selected food groups (mean value >0.5%)

| **Children**  **(n=1775)** | | | | | | | | | | | | |
| --- | --- | --- | --- | --- | --- | --- | --- | --- | --- | --- | --- | --- |
| **Food groups and categories** | **Medium** | | | | **High** | | | | **Medium/High** | | | |
|  | **Mean** | **Std** | **95%LCL*** | **95%UCL*** | **Mean** | **Std** | **95%LCL** | **95%UCL** | **Mean** | **Std** | **95%LCL** | **95%UCL** |
| Fruits and vegetables | 78.9 | 32.6 | 76.3 | 81.5 | 0.0 | 0.0 | 0.0 | 0.0 | 34.2 | 30.8 | 31.7 | 36.6 |
| Vegetables | 43.6 | 37.7 | 40.7 | 46.5 | 0.0 | 0.0 | 0.0 | 0.0 | 17.2 | 22.7 | 15.4 | 19.0 |
| Fruits | 35.3 | 38.1 | 32.2 | 38.4 | 0.0 | 0.0 | 0.0 | 0.0 | 17.0 | 24.1 | 15.1 | 18.8 |
| Fish/Meat/Eggs | 0.9 | 7.6 | 0.0 | 1.7 | 0.0 | 0.0 | 0.0 | 0.0 | 0.2 | 2.3 | 0.1 | 0.3 |
| Fish products | 0.7 | 7.3 | 0.1 | 1.5 | 0.0 | 0.0 | 0.0 | 0.0 | 0.2 | 2.0 | 0.1 | 0.3 |
| Meat | 0.2 | 2.4 | 0.0 | 0.3 | 0.0 | 0.0 | 0.0 | 0.0 | 0.1 | 1.2 | 0.0 | 0.1 |
| Dairy products | 14.6 | 28.7 | 12.6 | 16.7 | 99.8 | 2.6 | 99.7 | 100.0 | 63.1 | 31.6 | 60.7 | 65.5 |
| Milk & fresh dairy | 0.0 | 0.0 | 0.0 | 0.0 | 81.9 | 31.1 | 79.8 | 84.0 | 50.4 | 33.4 | 47.9 | 52.8 |
| Cheese | 14.6 | 28.7 | 12.6 | 16.7 | 17.9 | 31.0 | 15.8 | 20.0 | 12.7 | 21.5 | 11.4 | 14.1 |
| Mixed dishes | 4.9 | 16.8 | 3.8 | 6.0 | 0.0 | 0.0 | 0.0 | 0.0 | 2.2 | 9.4 | 1.6 | 2.8 |
| **Adults (n=2121)** | | | | | | | | | | | | |
| **Food groups and categories** | **Medium** | | | | **High** | | | | **Medium/High** | | | |
|  | **Mean** | **Std** | **95%LCL** | **95%UCL** | **Mean** | **Std** | **95%LCL** | **95%UCL** | **Mean** | **Std** | **95%LCL** | **95%UCL** |
| Fruits and vegetables | 75.4 | 32.4 | 73.5 | 77.4 | 0.0 | 1.1 | 0.0 | 0.0 | 40.8 | 30.1 | 38.7 | 42.8 |
| Vegetables | 50.4 | 36.2 | 48.2 | 52.5 | 0.0 | 1.1 | 0.0 | 0.0 | 25.5 | 25.0 | 24.1 | 26.9 |
| Fruits | 25.1 | 32.6 | 23.1 | 27.0 | 0.0 | 0.0 | 0.0 | 0.0 | 15.2 | 22.5 | 13.8 | 16.7 |
| Fish/Meat/Eggs | 1.8 | 10.3 | 1.1 | 2.6 | 0.0 | 0.0 | 0.0 | 0.0 | 1.1 | 7.1 | 0.5 | 1.7 |
| Fish products | 1.5 | 9.6 | 0.8 | 2.3 | 0.0 | 0.0 | 0.0 | 0.0 | 0.9 | 6.6 | 0.3 | 1.5 |
| Meat | 0.3 | 3.7 | 0.2 | 0.5 | 0.0 | 0.0 | 0.0 | 0.0 | 0.2 | 2.7 | 0.1 | 0.3 |
| Dairy products | 15.4 | 27.3 | 13.6 | 17.2 | 99.5 | 5.6 | 99.2 | 99.8 | 54.0 | 31.2 | 51.7 | 56.3 |
| Milk & fresh dairy | 0.0 | 0.0 | 0.0 | 0.0 | 66.7 | 38.8 | 63.7 | 69.7 | 35.0 | 30.9 | 32.8 | 37.1 |
| Cheese | 15.4 | 27.3 | 13.6 | 17.2 | 32.8 | 38.6 | 29.8 | 35.8 | 19.1 | 24.0 | 17.1 | 21.0 |
| Mixed dishes | 5.8 | 17.8 | 4.6 | 6.9 | 0.0 | 0.0 | 0.0 | 0.0 | 3.4 | 11.5 | 2.7 | 4.2 |

- LCL Lower confidence interval; UCL Upper confidence interval

Supplemental Table 4. Mean consumption (grams per day) of Medium, High and Medium/High foods by sociodemographic variables

| **Children (n=1775)** | | | | | | | | | | | | | | | | | | |
| --- | --- | --- | --- | --- | --- | --- | --- | --- | --- | --- | --- | --- | --- | --- | --- | --- | --- | --- |
|  | | **Medium foods consumers** | | | | | **High foods consumers** | | | | | | **Medium/High foods consumers** | | | | | |
|  |  | **Mean** | **Std** | **95%LCL*** | **95%UCL*** | **Pvalue** | **Mean** | **Std** | **95%LCL** | **95%UCL** | **Pvalue** | **Mean** | | **Std** | **95%LCL** | **95%UCL** | **Pvalue** |  |
| **All** | All | 69.2 | 78.7 | 64.3 | 74.1 |  | 92.0 | 77.3 | 86.6 | 97.4 |  | 161.2 | | 113.0 | 153.3 | 169.1 |  |  |
| **Gender** | Men | 70.1 | 79.0 | 63.4 | 76.8 | 0.696 | 95.0 | 78.5 | 87.4 | 102.7 | 0.218 | 165.1 | | 115.3 | 153.8 | 176.5 | 0.246 |  |
|  | Women | 68.2 | 78.4 | 61.5 | 74.9 |  | 88.8 | 75.9 | 82.0 | 95.7 |  | 157.1 | | 110.5 | 148.0 | 166.1 |  |  |
|  | Low | 58.2 | 67.0 | 51.6 | 64.8 | **<0.001** | 81.8 | 74.0 | 73.6 | 90.1 | **0.0052** | 140.0 | | 104.7 | 129.5 | 150.6 | **<0.001** |  |
|  | Middle | 71.3 | 77.4 | 63.3 | 79.4 |  | 99.0 | 81.0 | 89.4 | 108.6 |  | 170.3 | | 115.6 | 157.7 | 182.9 |  |  |
|  | High | 84.1 | 77.9 | 76.8 | 91.5 |  | 101.2 | 80.9 | 90.4 | 112.0 |  | 185.3 | | 110.9 | 171.7 | 198.9 |  |  |
|  | Inactive | 75.2 | 108.6 | 56.1 | 94.4 |  | 95.1 | 69.4 | 81.4 | 108.9 |  | 170.3 | | 125.6 | 147.4 | 193.3 |  |  |
| **Income per CU** | Missing value | 84.4 | 74.0 | 69.3 | 99.4 | **<0.001** | 99.6 | 83.1 | 77.3 | 121.8 | **0.0173** | 183.9 | | 108.7 | 157.7 | 210.1 | **<0.001** |  |
|  | <900 €/mo | 59.1 | 80.9 | 47.7 | 70.6 |  | 90.1 | 70.7 | 80.1 | 100.0 |  | 149.2 | | 104.5 | 133.5 | 164.9 |  |  |
|  | 900-1 340 €/mo | 62.1 | 72.7 | 53.5 | 70.7 |  | 82.5 | 77.0 | 73.0 | 91.9 |  | 144.6 | | 115.1 | 130.3 | 158.8 |  |  |
|  | 1 340-1 850 €/month | 76.8 | 81.5 | 67.8 | 85.9 |  | 103.1 | 81.4 | 93.9 | 112.2 |  | 179.9 | | 118.9 | 166.8 | 193.0 |  |  |
|  | >=1 850 €/month | 80.9 | 78.9 | 70.8 | 91.0 |  | 89.1 | 76.9 | 78.3 | 99.9 |  | 170.0 | | 108.9 | 155.7 | 184.3 |  |  |
| **Adults (n=2121)** | | | | | | | | | | | | | | | | | | |
|  |  | **Mean** | **Std** | **95%LCL** | **95%UCL** | **Pvalue** | **Mean** | **Std** | **95%LCL** | **95%UCL** | **Pvalue** | **Mean** | | **Std** | **95%LCL** | **95%UCL** | **Pvalue** |  |
| **All** | All | 117.7 | 120.6 | 110.6 | 124.8 |  | 94.8 | 81.0 | 89.0 | 100.6 |  | 212.5 | | 153.3 | 202.8 | 222.2 |  |  |
| **Gender** | Men | 126.5 | 131.0 | 115.1 | 138.0 | **0.0366** | 93.4 | 81.3 | 85.5 | 101.2 | 0.565 | 219.9 | | 161.7 | 206.0 | 233.7 | 0.157 |  |
|  | Women | 109.4 | 109.3 | 99.5 | 119.2 |  | 96.2 | 80.7 | 89.1 | 103.3 |  | 205.6 | | 144.6 | 191.8 | 219.3 |  |  |
| **Age** | 18-44 | 93.4 | 104.6 | 83.1 | 103.7 | **<0.001** | 92.4 | 81.4 | 84.0 | 100.8 | 0.0818 | 185.8 | | 144.6 | 170.7 | 200.9 | **<0.001** |  |
|  | 45-64 | 138.8 | 129.7 | 126.4 | 151.2 |  | 92.8 | 79.9 | 84.5 | 101.1 |  | 231.6 | | 159.1 | 216.2 | 247.0 |  |  |
|  | 65-79 | 137.2 | 128.2 | 121.5 | 153.0 |  | 105.3 | 81.4 | 95.4 | 115.3 |  | 242.6 | | 151.8 | 224.1 | 261.0 |  |  |
| **Socio professional category** | Low | 104.4 | 118.3 | 87.4 | 121.3 | 0.0936 | 88.0 | 82.6 | 77.5 | 98.5 | **0.0285** | 192.4 | | 163.5 | 169.2 | 215.6 | **0.0075** |  |
|  | Middle | 119.4 | 110.7 | 103.3 | 135.4 |  | 89.6 | 78.7 | 78.8 | 100.3 |  | 209.0 | | 140.0 | 189.7 | 228.2 |  |  |
|  | High | 136.6 | 122.9 | 120.0 | 153.3 |  | 111.1 | 82.2 | 100.4 | 121.8 |  | 247.7 | | 147.8 | 228.3 | 267.2 |  |  |
|  | Inactive | 119.0 | 126.2 | 107.3 | 130.7 |  | 96.5 | 79.6 | 85.5 | 107.4 |  | 215.5 | | 152.7 | 200.0 | 230.9 |  |  |
| **Income per CU** | <900 €/month | 93.8 | 114.7 | 75.3 | 112.3 | **0.0132** | 80.3 | 82.3 | 66.4 | 94.2 | **0.0439** | 174.1 | | 161.1 | 148.5 | 199.8 | **0.0017** |  |
|  | 900-1 340 €/month | 124.4 | 126.4 | 108.0 | 140.8 |  | 93.2 | 86.0 | 82.3 | 104.0 |  | 217.6 | | 156.6 | 199.4 | 235.7 |  |  |
|  | 1 340-1 850 €/month | 123.5 | 130.8 | 109.1 | 137.9 |  | 97.5 | 77.4 | 86.9 | 108.1 |  | 221.0 | | 153.5 | 203.2 | 238.8 |  |  |
|  | >=1 850 €/month | 131.6 | 112.5 | 119.6 | 143.7 |  | 103.5 | 79.0 | 96.1 | 111.0 |  | 235.2 | | 142.6 | 220.1 | 250.3 |  |  |
|  | Missing value | 103.5 | 106.1 | 86.7 | 120.3 |  | 106.1 | 72.2 | 91.6 | 120.5 |  | 209.6 | | 132.5 | 188.2 | 231.0 |  |  |

- LCL Lower confidence interval; UCL Upper confidence interval

Supplemental Table 5. Mean total consumption (in kcal per day) and mean consumption from Medium/High foods by sociodemographic variables for children (n=1775) and adults (n=2121)

|  | **Children kcal total** | | | | **Children kcal**  **From Medium/High foods** | | | **Adults kcal total** | | | **Adults kcal**  **From Medium/High foods** | | |
| --- | --- | --- | --- | --- | --- | --- | --- | --- | --- | --- | --- | --- | --- |
|  | **Mean** | **95%LCL*** | **95%UCL*** | **Mean** | | **95%LCL** | **95%UCL** | **Mean** | **95%LCL** | **95%UCL** | **Mean** | **95%LCL** | **95%UCL** |
| All | 1833 | 1781 | 1885 | 159.9 | | 151.8 | 167.9 | 2009 | 1958 | 2060 | 223.7 | 212.2 | 235.2 |
| Sex |  |  |  |  | |  |  |  |  |  |  |  |  |
| Male | 1945 | 1903 | 1988 | 164.9 | | 153.0 | 176.8 | 2293 | 2212 | 2374 | 249.4 | 229.2 | 269.6 |
| Female | 1715 | 1613 | 1818 | 154.6 | | 145.1 | 164.1 | 1742 | 1690 | 1793 | 199.5 | 187.0 | 212.0 |
| Age |  |  |  |  | |  |  |  |  |  |  |  |  |
| 18-44y |  |  |  |  | |  |  | 2098 | 2012 | 2184 | 211.0 | 189.2 | 232.9 |
| 45-64 y |  |  |  |  | |  |  | 1997 | 1909 | 2084 | 227.4 | 212.4 | 242.4 |
| 65-79 y |  |  |  |  | |  |  | 1802 | 1731 | 1873 | 249.1 | 233.2 | 265.0 |
| **Socio professional category** |  |  |  |  | |  |  |  |  |  |  |  |  |
| Low | 1887 | 1779 | 1995 | 143.3 | | 131.6 | 155.0 | 2039 | 1919 | 2158 | 208.1 | 174.9 | 241.3 |
| Middle | 1794 | 1738 | 1850 | 170.6 | | 157.1 | 184.2 | 2033 | 1915 | 2151 | 212.5 | 194.8 | 230.2 |
| High | 1788 | 1725 | 1851 | 178.5 | | 162.0 | 195.1 | 2159 | 2075 | 2244 | 265.3 | 242.3 | 288.4 |
| Inactive | 1816 | 1729 | 1902 | 159.5 | | 142.2 | 176.8 | 1907 | 1830 | 1984 | 225.1 | 207.1 | 243.2 |
| Income per CU |  |  |  |  | |  |  |  |  |  |  |  |  |
| <900 | 1895 | 1731 | 2060 | 148.6 | | 133.6 | 163.7 | 1979 | 1872 | 2088 | 189.7 | 151.4 | 227.9 |
| 900-1340 | 1754 | 1698 | 1808 | 142.6 | | 127.7 | 157.4 | 2001 | 1880 | 2121 | 221.8 | 203.6 | 240.0 |
| 1340-1850 | 1836 | 1776 | 1896 | 179.1 | | 166.5 | 191.8 | 2051 | 1944 | 2158 | 233.9 | 215.3 | 252.4 |
| >1850 | 1833 | 1758 | 1908 | 164.6 | | 149.7 | 179.5 | 2028 | 1951 | 2105 | 245.3 | 231.7 | 258.9 |
| Missing | 1871 | 1725 | 2018 | 187.4 | | 150.1 | 224.8 | 1924 | 1763 | 2085 | 226.7 | 200.3 | 253.2 |

- LCL Lower confidence interval; UCL Upper confidence interval

Supplemental Table 6. Contribution (%) of Medium, High, Medium/High foods to total nutrient intakes

| Children (n=1775) | | | | | | | | | | | | |
| --- | --- | --- | --- | --- | --- | --- | --- | --- | --- | --- | --- | --- |
|  | **Medium** | | | | **High** | | | | **Medium/High** | | | |
| **Nutrient** | **Mean** | **Std** | **95%LCL** | **95%UCL** | **Mean** | **Std** | **95%LCL** | **95%UCL** | **Mean** | **Std** | **95%LCL** | **95%UCL** |
| **Energy** | 2.7 | 3.1 | 2.4 | 2.9 | 5.9 | 4.7 | 5.5 | 6.2 | 8.5 | 5.7 | 8.1 | 9.0 |
| **Carbohydrates** | 2.3 | 3.3 | 2.1 | 2.5 | 4.2 | 4.1 | 3.9 | 4.4 | 6.4 | 5.2 | 6.1 | 6.8 |
| **Protein** | 2.7 | 3.9 | 2.4 | 3.0 | 8.0 | 6.6 | 7.6 | 8.5 | 10.7 | 7.8 | 10.1 | 11.4 |
| **Fibre** | 6.2 | 7.2 | 5.7 | 6.7 | 0.6 | 0.9 | 0.6 | 0.7 | 6.9 | 7.3 | 6.4 | 7.4 |
| **Lipids** | 2.7 | 4.6 | 2.4 | 3.1 | 7.6 | 6.8 | 7.1 | 8.0 | 10.3 | 8.5 | 9.6 | 10.9 |
| **SFA, g/d** | 3.2 | 6.0 | 2.7 | 3.7 | 11.1 | 9.7 | 10.5 | 11.8 | 14.3 | 11.7 | 13.4 | 15.2 |
| **EPA and DHA** | 1.5 | 5.8 | 1.1 | 1.9 | 3.9 | 5.1 | 3.5 | 4.2 | 5.4 | 7.8 | 4.9 | 5.9 |
| **Linoleic fatty acid** | 2.0 | 3.6 | 1.8 | 2.3 | 1.5 | 1.6 | 1.4 | 1.6 | 3.5 | 4.0 | 3.2 | 3.8 |
| **α-linolenic fatty acid** | 3.4 | 5.2 | 3.0 | 3.7 | 4.1 | 4.5 | 3.8 | 4.4 | 7.5 | 7.2 | 7.0 | 8.0 |
| **Oleic fatty acid** | 2.1 | 4.1 | 1.8 | 2.4 | 5.0 | 4.9 | 4.7 | 5.4 | 7.2 | 6.6 | 6.7 | 7.7 |
| **Palmitic acid** | 3.1 | 5.7 | 2.7 | 3.6 | 9.5 | 8.6 | 8.9 | 10.1 | 12.6 | 10.7 | 11.8 | 13.4 |
| **Stearic fatty acid** | 2.7 | 5.2 | 2.3 | 3.2 | 8.4 | 7.9 | 7.9 | 9.0 | 11.2 | 9.8 | 10.4 | 11.9 |
| **Myristic fatty acid** | 4.7 | 9.2 | 4.1 | 5.4 | 17.2 | 14.6 | 16.1 | 18.2 | 21.9 | 17.2 | 20.6 | 23.2 |
| **Lauric fatty acid** | 3.9 | 8.1 | 3.3 | 4.5 | 13.9 | 13.0 | 13.0 | 14.9 | 17.9 | 15.4 | 16.7 | 19.0 |
| **Total sugar** | 3.9 | 5.8 | 3.6 | 4.3 | 8.2 | 8.4 | 7.7 | 8.8 | 12.2 | 9.9 | 11.5 | 12.8 |
| **Free sugar** | 0.0 | 0.8 | 0.0 | 0.1 | 6.0 | 8.4 | 5.4 | 6.6 | 6.1 | 8.5 | 5.5 | 6.6 |
| **Calcium** | 4.5 | 7.0 | 4.1 | 5.0 | 17.8 | 13.3 | 16.8 | 18.7 | 22.3 | 14.9 | 21.2 | 23.4 |
| **Iron** | 2.1 | 2.8 | 2.0 | 2.3 | 2.1 | 2.2 | 1.9 | 2.2 | 4.2 | 3.6 | 4.0 | 4.5 |
| **Iodine** | 2.4 | 4.0 | 2.1 | 2.6 | 9.9 | 9.3 | 9.3 | 10.6 | 12.3 | 10.2 | 11.6 | 13.0 |
| **Magnesium** | 3.6 | 3.9 | 3.3 | 3.9 | 5.1 | 4.2 | 4.8 | 5.4 | 8.7 | 5.8 | 8.3 | 9.2 |
| **Potassium** | 5.4 | 6.0 | 5.0 | 5.8 | 5.6 | 5.0 | 5.3 | 6.0 | 11.0 | 7.8 | 10.4 | 11.6 |
| **Sodium** | 1.9 | 3.5 | 1.7 | 2.2 | 3.9 | 3.9 | 3.6 | 4.1 | 5.8 | 5.4 | 5.5 | 6.2 |
| **Phosphorus** | 3.6 | 4.6 | 3.3 | 3.9 | 10.5 | 8.1 | 9.9 | 11.0 | 14.0 | 9.4 | 13.3 | 14.8 |
| **Selenium** | 3.4 | 4.5 | 3.1 | 3.7 | 4.4 | 5.3 | 4.0 | 4.7 | 7.8 | 7.0 | 7.3 | 8.2 |
| **Zinc** | 3.3 | 5.2 | 2.9 | 3.7 | 8.0 | 7.0 | 7.5 | 8.5 | 11.3 | 8.8 | 10.7 | 12.0 |
| **Thiamin** | 2.3 | 2.7 | 2.1 | 2.4 | 5.8 | 6.3 | 5.4 | 6.2 | 8.1 | 6.8 | 7.6 | 8.5 |
| **Riboflavin** | 2.8 | 3.8 | 2.5 | 3.1 | 14.3 | 11.4 | 13.4 | 15.1 | 17.1 | 12.0 | 16.2 | 18.0 |
| **Niacin** | 2.7 | 4.1 | 2.4 | 2.9 | 1.2 | 1.4 | 1.2 | 1.3 | 3.9 | 4.4 | 3.6 | 4.2 |
| **Pantothenic acid** | 2.8 | 3.4 | 2.6 | 3.0 | 8.8 | 8.1 | 8.3 | 9.4 | 11.6 | 8.7 | 11.0 | 12.3 |
| **Vitamin B6** | 4.0 | 4.7 | 3.6 | 4.3 | 3.5 | 3.2 | 3.3 | 3.7 | 7.4 | 5.8 | 7.0 | 7.9 |
| **Folate** | 6.2 | 7.0 | 5.8 | 6.7 | 7.4 | 6.3 | 6.9 | 7.8 | 13.6 | 9.2 | 13.0 | 14.3 |
| **Vitamin B12** | 2.4 | 5.9 | 2.0 | 2.8 | 8.9 | 8.3 | 8.3 | 9.5 | 11.3 | 10.1 | 10.6 | 12.0 |
| **Vitamin A** | 13.8 | 16.7 | 12.6 | 15.0 | 7.7 | 7.8 | 7.2 | 8.2 | 21.5 | 17.7 | 20.3 | 22.8 |
| **Vitamin C** | 10.8 | 14.6 | 9.9 | 11.7 | 0.4 | 0.6 | 0.4 | 0.4 | 11.2 | 14.6 | 10.3 | 12.1 |
| **Vitamin D** | 1.7 | 3.4 | 1.5 | 2.0 | 20.7 | 17.3 | 19.5 | 21.8 | 22.4 | 17.4 | 21.2 | 23.6 |
| **Vitamin E** | 5.0 | 6.6 | 4.5 | 5.4 | 1.4 | 1.4 | 1.3 | 1.5 | 6.4 | 6.8 | 5.9 | 6.8 |
| **Adults (n=2121)** | | | | | | | | | | | | |
|  | **Medium** | | | | **High** | | | | **Medium/High** | | | |
| **Nutrient** | **Mean** | **Std** | **95%LCL** | **95%UCL** | **Mean** | **Std** | **95%LCL** | **95%UCL** | **Mean** | **Std** | **95%LCL** | **95%UCL** |
| **Energy** | 4.2 | 4.3 | 3.9 | 4.5 | 6.5 | 5.2 | 6.2 | 6.9 | 10.7 | 6.8 | 10.3 | 11.2 |
| **Carbohydrates** | 3.4 | 4.8 | 3.1 | 3.7 | 3.3 | 3.9 | 3.1 | 3.6 | 6.7 | 6.3 | 6.3 | 7.1 |
| **Protein** | 4.6 | 5.4 | 4.2 | 4.9 | 9.0 | 7.2 | 8.6 | 9.4 | 13.6 | 8.8 | 13.0 | 14.1 |
| **Fibre** | 8.5 | 9.0 | 7.9 | 9.0 | 0.5 | 1.1 | 0.5 | 0.6 | 9.0 | 9.0 | 8.5 | 9.5 |
| **Lipids** | 4.7 | 6.3 | 4.3 | 5.1 | 9.8 | 8.8 | 9.2 | 10.4 | 14.6 | 10.5 | 13.8 | 15.3 |
| **SFA, g/d** | 5.7 | 8.2 | 5.2 | 6.2 | 14.9 | 12.8 | 14.1 | 15.8 | 20.6 | 14.4 | 19.7 | 21.6 |
| **EPA and DHA** | 3.2 | 9.4 | 2.5 | 3.8 | 4.4 | 6.3 | 4.0 | 4.8 | 7.6 | 11.2 | 6.9 | 8.4 |
| **Linoleic fatty acid** | 3.5 | 6.0 | 3.2 | 3.8 | 2.0 | 2.1 | 1.8 | 2.1 | 5.5 | 6.4 | 5.1 | 5.9 |
| **α-linolenic fatty acid** | 5.4 | 7.0 | 4.9 | 5.9 | 5.1 | 5.2 | 4.7 | 5.4 | 10.5 | 8.8 | 9.8 | 11.1 |
| **Oleic fatty acid** | 3.8 | 6.1 | 3.5 | 4.2 | 6.7 | 6.4 | 6.2 | 7.1 | 10.5 | 8.7 | 9.9 | 11.1 |
| **Palmitic acid** | 5.4 | 7.6 | 5.0 | 5.9 | 13.0 | 11.5 | 12.2 | 13.7 | 18.4 | 13.1 | 17.5 | 19.3 |
| **Stearic fatty acid** | 5.0 | 7.5 | 4.6 | 5.5 | 12.0 | 10.9 | 11.2 | 12.7 | 17.0 | 12.8 | 16.1 | 17.8 |
| **Myristic fatty acid** | 8.4 | 12.8 | 7.6 | 9.3 | 22.6 | 18.7 | 21.4 | 23.9 | 31.1 | 20.4 | 29.7 | 32.4 |
| **Lauric fatty acid** | 7.3 | 11.7 | 6.6 | 8.0 | 19.8 | 17.6 | 18.7 | 21.0 | 27.2 | 19.7 | 25.9 | 28.4 |
| **Total sugar** | 6.7 | 9.6 | 6.0 | 7.3 | 7.4 | 8.6 | 6.9 | 7.9 | 14.1 | 12.7 | 13.3 | 14.9 |
| **Free sugar** | 0.1 | 1.3 | 0.1 | 0.2 | 5.5 | 11.5 | 4.8 | 6.3 | 5.6 | 11.6 | 4.9 | 6.4 |
| **Calcium** | 8.3 | 9.7 | 7.6 | 8.9 | 21.2 | 15.4 | 20.2 | 22.2 | 29.5 | 16.8 | 28.4 | 30.5 |
| **Iron** | 4.2 | 4.8 | 3.9 | 4.4 | 2.2 | 2.4 | 2.0 | 2.3 | 6.3 | 5.6 | 6.0 | 6.7 |
| **Iodine** | 4.5 | 6.4 | 4.1 | 5.0 | 11.0 | 10.0 | 10.3 | 11.7 | 15.5 | 11.6 | 14.7 | 16.4 |
| **Magnesium** | 4.8 | 4.5 | 4.5 | 5.0 | 4.4 | 3.6 | 4.2 | 4.7 | 9.2 | 5.9 | 8.8 | 9.6 |
| **Potassium** | 7.9 | 7.3 | 7.5 | 8.3 | 4.8 | 4.3 | 4.5 | 5.1 | 12.7 | 8.6 | 12.1 | 13.2 |
| **Sodium** | 3.3 | 4.9 | 3.1 | 3.6 | 4.6 | 4.7 | 4.3 | 4.9 | 8.0 | 6.6 | 7.6 | 8.3 |
| **Phosphorus** | 6.1 | 6.4 | 5.7 | 6.5 | 12.1 | 9.2 | 11.5 | 12.7 | 18.2 | 10.8 | 17.5 | 18.9 |
| **Selenium** | 4.4 | 5.1 | 4.0 | 4.8 | 3.1 | 3.1 | 2.9 | 3.2 | 7.5 | 6.0 | 7.1 | 7.9 |
| **Zinc** | 6.1 | 9.0 | 5.5 | 6.8 | 9.1 | 8.0 | 8.7 | 9.6 | 15.3 | 11.6 | 14.5 | 16.1 |
| **Thiamin** | 4.1 | 4.3 | 3.8 | 4.3 | 4.8 | 5.3 | 4.5 | 5.2 | 8.9 | 6.9 | 8.5 | 9.3 |
| **Riboflavin** | 5.0 | 5.4 | 4.6 | 5.3 | 15.0 | 11.5 | 14.3 | 15.8 | 20.0 | 12.3 | 19.2 | 20.8 |
| **Niacin** | 3.6 | 4.4 | 3.3 | 3.8 | 1.3 | 1.4 | 1.2 | 1.4 | 4.8 | 4.7 | 4.6 | 5.1 |
| **Pantothenic acid** | 5.1 | 5.0 | 4.8 | 5.5 | 8.4 | 7.3 | 7.9 | 8.8 | 13.5 | 8.8 | 12.9 | 14.1 |
| **Vitamin B6** | 6.4 | 6.4 | 6.0 | 6.8 | 3.9 | 3.6 | 3.7 | 4.2 | 10.3 | 7.6 | 9.8 | 10.8 |
| **Folate** | 10.9 | 9.5 | 10.3 | 11.5 | 7.9 | 6.8 | 7.5 | 8.3 | 18.8 | 11.6 | 18.1 | 19.5 |
| **Vitamin B12** | 5.1 | 11.2 | 4.3 | 6.0 | 10.4 | 10.3 | 9.8 | 11.0 | 15.6 | 14.4 | 14.6 | 16.5 |
| **Vitamin A** | 19.8 | 18.4 | 18.6 | 21.0 | 8.3 | 8.4 | 7.8 | 8.8 | 28.1 | 19.5 | 26.8 | 29.4 |
| **Vitamin C** | 15.0 | 17.1 | 14.1 | 16.0 | 0.4 | 0.7 | 0.3 | 0.4 | 15.4 | 17.1 | 14.5 | 16.3 |
| **Vitamin D** | 4.0 | 7.1 | 3.4 | 4.5 | 18.8 | 17.2 | 17.6 | 19.9 | 22.7 | 18.0 | 21.5 | 24.0 |
| **Vitamin E** | 7.5 | 8.6 | 7.0 | 8.0 | 1.8 | 1.9 | 1.7 | 1.9 | 9.3 | 8.9 | 8.7 | 9.8 |

- LCL Lower confidenced interval; UCL Upper confidence interval

Supplemental table 7. Mean intakes (g/day by food groups and subgroups and tertile of Medium/High consumption among adults, and adjusted means (on total energy intakes, IUC, PCS and BMI).

| **Children (n=1775)** | | | | | | | | | | | | | | | | | | |
| --- | --- | --- | --- | --- | --- | --- | --- | --- | --- | --- | --- | --- | --- | --- | --- | --- | --- | --- |
|  | **Tertile 1 [0,98]** | | | | | **Tertile 2 [98,191]** | | | | | **Tertile 3 ≥191** | | | | |  |  |  |
|  | **Mean** | **Std** | **95%**  **LCL** | **95%**  **UCL** | **Mean**  **adj** | **Mean** | **Std** | **95%**  **LCL** | **95%**  **UCL** | **Mean**  **adj** | **Mean** | **Std** | **95%**  **LCL** | **95%**  **UCL** | **Mean**  **adj** | **pval** | **Pval1** | **Pval2** |
| **Fruits and vegetables** | 236.0 | 182.1 | 216.7 | 255.3 | 262.4 | 295.8 | 167.5 | 278.1 | 313.6 | 311.4 | 485.8 | 241.8 | 460.8 | 510.7 | 491.8 | **<0.001** | **<0.001** | **<0.001** |
| Vegetables | 56.8 | 65.0 | 49.4 | 64.2 | 67.9 | 89.1 | 69.8 | 81.9 | 96.4 | 96.7 | 152.3 | 119.4 | 142.0 | 162.7 | 157.2 | **<0.001** | **<0.001** | **<0.001** |
| Fruits | 84.6 | 94.1 | 74.6 | 94.6 | 92.1 | 116.2 | 93.2 | 106.9 | 125.5 | 121.3 | 225.3 | 169.1 | 207.8 | 242.8 | 228.2 | **<0.001** | **<0.001** | **<0.001** |
| Nuts | 0.3 | 2.1 | 0.1 | 0.4 | 0.3 | 0.6 | 2.9 | 0.4 | 0.8 | 0.5 | 0.9 | 4.2 | 0.6 | 1.3 | 0.8 | **0.0014** | **0.0056** | **0.0434** |
| Fruit juices | 94.3 | 125.5 | 80.3 | 108.3 | 102.2 | 89.9 | 109.8 | 79.2 | 100.6 | 92.8 | 107.2 | 116.5 | 94.4 | 120.0 | 105.6 | 0.0674 | 0.314 | 0.256 |
| **Starches** | 132.6 | 91.6 | 121.7 | 143.4 | 149.0 | 132.7 | 94.8 | 118.8 | 146.7 | 140.1 | 152.0 | 106.5 | 140.0 | 164.0 | 148.7 | 0.0561 | 0.306 | 0.302 |
| Whole grains | 5.5 | 16.7 | 4.0 | 7.0 | 5.6 | 6.2 | 15.5 | 4.5 | 8.0 | 5.5 | 10.5 | 24.2 | 7.3 | 13.6 | 9.3 | **0.0145** | **0.0348** | 0.0614 |
| Refined grains | 127.0 | 89.7 | 116.4 | 137.7 | 143.4 | 126.5 | 92.6 | 112.6 | 140.4 | 134.6 | 141.5 | 107.3 | 129.4 | 153.7 | 139.4 | 0.192 | 0.313 | 0.366 |
| **Legumes and tubers** | 40.3 | 47.9 | 34.8 | 45.8 | 44.4 | 42.9 | 50.8 | 34.9 | 50.9 | 45.2 | 36.9 | 42.4 | 33.2 | 40.6 | 36.6 | 0.257 | **0.0047** | **0.0123** |
| Potatoes and tubers | 36.0 | 44.5 | 31.1 | 40.9 | 39.3 | 39.4 | 50.6 | 31.2 | 47.7 | 41.0 | 31.0 | 41.4 | 27.4 | 34.7 | 30.1 | 0.0729 | **<0.001** | **0.0031** |
| Legumes | 4.3 | 18.2 | 2.0 | 6.6 | 5.1 | 3.4 | 10.9 | 2.5 | 4.4 | 4.2 | 5.9 | 16.2 | 4.3 | 7.4 | 6.5 | **0.0265** | **0.0426** | **0.0355** |
| **Meat/Fish/Eggs** | 99.3 | 57.5 | 91.8 | 106.9 | 98.4 | 102.3 | 58.7 | 94.7 | 109.8 | 98.3 | 113.0 | 66.5 | 105.6 | 120.4 | 107.8 | **0.0309** | 0.451 | 0.146 |
| Fish and shellfish | 15.3 | 24.6 | 12.5 | 18.2 | 14.2 | 17.5 | 25.0 | 14.9 | 20.0 | 15.6 | 20.1 | 32.2 | 16.7 | 23.4 | 17.9 | 0.0983 | 0.235 | 0.27 |
| Meat | 74.3 | 48.7 | 69.1 | 79.6 | 77.0 | 78.0 | 54.8 | 70.8 | 85.2 | 78.2 | 83.7 | 57.9 | 76.9 | 90.5 | 82.5 | 0.0808 | 0.687 | 0.35 |
| Eggs | 9.7 | 28.7 | 3.5 | 15.8 | 7.3 | 6.8 | 16.5 | 5.4 | 8.3 | 4.6 | 9.2 | 18.2 | 7.6 | 10.9 | 7.4 | 0.17 | 0.198 | 0.137 |
| **Dairy products** | 283.7 | 213.6 | 260.3 | 307.2 | 278.2 | 425.8 | 220.4 | 397.4 | 454.3 | 416.7 | 560.7 | 267.8 | 531.4 | 590.0 | 545.2 | **<0.001** | **<0.001** | **<0.001** |
| Milk and fresh dairy | 263.6 | 214.6 | 240.3 | 286.8 | 253.7 | 393.5 | 218.0 | 366.1 | 420.8 | 381.2 | 518.9 | 263.8 | 491.1 | 546.8 | 502.5 | **<0.001** | **<0.001** | **<0.001** |
| Cheese | 20.2 | 28.1 | 16.8 | 23.5 | 24.6 | 32.4 | 36.7 | 28.4 | 36.4 | 35.4 | 41.7 | 49.2 | 35.1 | 48.3 | 42.7 | **<0.001** | **<0.001** | **<0.001** |
| **Mixed dishes** | 182.2 | 129.6 | 168.2 | 196.1 | 195.2 | 185.0 | 138.2 | 166.6 | 203.5 | 188.6 | 181.4 | 144.8 | 166.2 | 196.7 | 173.2 | 0.961 | 0.166 | 0.107 |
| **Sweeta and fats** | 174.7 | 92.8 | 165.0 | 184.3 | 173.0 | 163.7 | 95.9 | 145.3 | 182.0 | 151.8 | 151.9 | 72.5 | 144.5 | 159.3 | 129.0 | **0.0019** | **<0.001** | **<0.001** |
| Sweetened products | 154.3 | 81.5 | 146.2 | 162.4 | 150.5 | 144.9 | 84.5 | 129.8 | 159.9 | 131.7 | 134.3 | 65.4 | 127.7 | 140.9 | 112.1 | **0.0013** | **<0.001** | **<0.001** |
| Salty and fat products | 20.3 | 36.4 | 16.3 | 24.4 | 22.5 | 18.8 | 30.4 | 14.2 | 23.4 | 20.1 | 17.6 | 29.9 | 14.2 | 21.0 | 16.9 | 0.588 | 0.0892 | 0.0818 |
| **Beverages** | 713.5 | 393.5 | 666.8 | 760.2 | 844.4 | 705.0 | 395.8 | 662.9 | 747.1 | 812.5 | 824.0 | 500.2 | 762.8 | 885.1 | 895.6 | **0.0069** | 0.0733 | 0.0796 |
| Water | 541.3 | 384.9 | 492.4 | 590.3 | 641.0 | 570.4 | 364.9 | 528.7 | 612.1 | 652.0 | 688.0 | 450.0 | 635.6 | 740.3 | 745.7 | **<0.001** | **0.0043** | **0.0068** |
| Hot drinks | 18.4 | 70.9 | 11.2 | 25.6 | 17.8 | 15.7 | 66.5 | 9.3 | 22.1 | 14.4 | 20.1 | 74.0 | 13.2 | 26.9 | 18.6 | 0.637 | 0.672 | 0.65 |
| Sweetened drinks | 153.8 | 195.7 | 129.7 | 177.8 | 185.7 | 118.9 | 156.3 | 98.3 | 139.6 | 146.0 | 115.9 | 185.4 | 89.1 | 142.8 | 131.3 | 0.0555 | **<0.001** | **<0.001** |
| **Fats and oils** | 13.0 | 14.8 | 11.3 | 14.7 | 15.8 | 15.0 | 16.6 | 12.7 | 17.3 | 16.8 | 17.6 | 18.7 | 15.9 | 19.2 | 18.1 | **<0.001** | 0.06 | 0.0879 |
| Vegetable fats | 3.5 | 5.7 | 2.8 | 4.1 | 4.5 | 5.0 | 7.0 | 4.3 | 5.7 | 5.8 | 8.0 | 11.2 | 6.9 | 9.1 | 8.5 | **<0.001** | **<0.001** | **<0.001** |
| Animal fats | 5.0 | 8.0 | 3.9 | 6.1 | 5.3 | 5.1 | 6.7 | 4.4 | 5.8 | 5.2 | 5.0 | 7.4 | 4.3 | 5.7 | 4.8 | 0.981 | 0.766 | 0.704 |
| Sauces | 4.6 | 11.9 | 3.4 | 5.8 | 6.0 | 4.9 | 13.3 | 3.0 | 6.8 | 5.8 | 4.6 | 13.0 | 3.3 | 5.8 | 4.8 | 0.953 | 0.438 | 0.419 |
| **Adults (n=2121)** | | | | | | | | | | | | | | | | | | |
|  | **Tertile 1 [0,133]** | | | | | **Tertile 2 [133,248]** | | | | | **Tertile 3 ≥248** | | | | |  |  |  |
|  | **Mean** | **Std** | **95%**  **LCL** | **95%**  **UCL** | **Mean**  **adj** | **Mean** | **Std** | **95%**  **LCL** | **95%**  **UCL** | **Mean**  **adj** | **Mean** | **Std** | **95%**  **LCL** | **95%**  **UCL** | **Mean**  **adj** | **pval** | **Pval1** | **Pval2** |
| **Fruits and vegetables** | 240.8 | 177.7 | 223 | 258.6 | 216.1 | 382.9 | 192 | 361.2 | 404.7 | 341.3 | 681.5 | 317.6 | 646.6 | 716.3 | 630.2 | **<0.001** | **<0.001** | **<0.001** |
| Vegetables | 96.3 | 93.4 | 87.9 | 104.6 | 75.7 | 161.3 | 120 | 147.1 | 175.5 | 137.5 | 292.5 | 193.6 | 274.3 | 310.7 | 268.7 | **<0.001** | **<0.001** | **<0.001** |
| Fruits | 86.1 | 99.2 | 75 | 97.1 | 73.5 | 159.1 | 129.9 | 146.3 | 171.8 | 136.4 | 315.8 | 247.1 | 284.4 | 347.1 | 287.5 | **<0.001** | **<0.001** | **<0.001** |
| Nuts | 2.4 | 7.8 | 1.4 | 3.4 | 2.1 | 2.2 | 7 | 1.6 | 2.8 | 1.6 | 2.7 | 8.2 | 1.9 | 3.4 | 1.9 | 0.693 | 0.718 | 0.704 |
| Fruit juices | 56.1 | 93 | 46.7 | 65.5 | 64.7 | 60.4 | 99.4 | 49.3 | 71.4 | 65.8 | 70.5 | 101.3 | 58.9 | 82.1 | 72.1 | 0.142 | 0.5 | 0.574 |
| **Starches** | 165.6 | 112.6 | 151 | 180.2 | 184.3 | 186.4 | 124 | 166.9 | 205.8 | 184.3 | 184.1 | 117.1 | 172.8 | 195.5 | 173.6 | 0.114 | 0.333 | 0.291 |
| Whole grains | 12 | 28.8 | 9.4 | 14.6 | 13.1 | 17.3 | 37 | 13.5 | 21 | 16.2 | 24.2 | 44.7 | 20.4 | 28 | 22.9 | **<0.001** | **<0.001** | **<0.001** |
| Refined grains | 153.6 | 113.4 | 138.7 | 168.4 | 171.1 | 169.1 | 124.4 | 148.4 | 189.9 | 168.1 | 159.9 | 118.3 | 148.9 | 170.9 | 150.7 | 0.51 | **0.0256** | **0.022** |
| **Legumes and tubers** | 58.9 | 81.9 | 45.8 | 72 | 61.4 | 52.4 | 78.4 | 41 | 63.8 | 50.1 | 49.3 | 70.6 | 41.7 | 56.8 | 42.3 | 0.329 | **0.0046** | **0.0049** |
| Potatoes and tubers | 51.9 | 79.3 | 38.6 | 65.2 | 54.2 | 45.2 | 76.1 | 34.1 | 56.2 | 43.4 | 40.4 | 63.4 | 34.1 | 46.8 | 34.5 | 0.16 | **0.0107** | **0.0048** |
| Legumes | 7 | 22.5 | 4.1 | 9.9 | 7.2 | 7.3 | 23.9 | 4.1 | 10.4 | 6.8 | 8.8 | 28 | 4.7 | 13 | 7.8 | 0.801 | 0.782 | 0.884 |
| **Meat/Fish/Eggs** | 134.3 | 84.5 | 121.9 | 146.8 | 121.8 | 142.4 | 83.9 | 130.4 | 154.4 | 120.6 | 151.4 | 88.2 | 141.9 | 160.8 | 125.4 | 0.151 | 0.838 | 0.802 |
| Fish and shellfish | 23.7 | 37.7 | 19.3 | 28 | 23.5 | 24.4 | 37.1 | 19.1 | 29.6 | 22.9 | 35.6 | 49.5 | 29.9 | 41.2 | 34.1 | **0.0039** | **0.0082** | **0.0131** |
| Meat | 100.1 | 75.8 | 90.3 | 109.9 | 87.7 | 107.9 | 79.3 | 96.7 | 119.1 | 87.7 | 102.8 | 70.2 | 94.5 | 111.2 | 78.3 | 0.58 | 0.303 | 0.277 |
| Eggs | 10.6 | 23.3 | 7.9 | 13.3 | 10.7 | 10.1 | 22.1 | 8 | 12.2 | 10 | 12.9 | 24.4 | 10.8 | 15.1 | 13 | 0.17 | 0.16 | 0.168 |
| **Dairy products** | 177.2 | 174 | 157 | 197.4 | 192.9 | 346.2 | 240.2 | 316.2 | 376.1 | 347.2 | 454.1 | 290 | 414.7 | 493.6 | 444.3 | **<0.001** | **<0.001** | **<0.001** |
| Milk & fresh dairy | 141.5 | 171 | 121.6 | 161.3 | 156 | 279.2 | 242.6 | 248.8 | 309.6 | 287.4 | 380.1 | 291.6 | 341.9 | 418.4 | 381.7 | **<0.001** | **<0.001** | **<0.001** |
| Cheese | 35.7 | 42.9 | 30.8 | 40.5 | 36.9 | 67 | 60.9 | 60.4 | 73.7 | 59.8 | 74 | 67.8 | 67.7 | 80.3 | 62.6 | **<0.001** | **<0.001** | **<0.001** |
| **Mixed dishes** | 239.9 | 182.4 | 219.5 | 260.4 | 246.3 | 277.5 | 221 | 252.7 | 302.3 | 269 | 265.9 | 222.9 | 238.9 | 292.8 | 249.8 | 0.0829 | 0.373 | 0.391 |
| **Sweets and fats** | 132.5 | 101.2 | 119.6 | 145.5 | 155.7 | 135.7 | 97.1 | 125.7 | 145.7 | 139.5 | 135.3 | 108.9 | 122.9 | 147.6 | 130.4 | 0.921 | **0.0025** | **0.0012** |
| Sweetened products | 110.4 | 87.4 | 99.1 | 121.6 | 128.8 | 114.6 | 87.9 | 104.8 | 124.4 | 114.6 | 113.1 | 92.6 | 103.6 | 122.6 | 105.1 | 0.857 | **<0.001** | **<0.001** |
| Salty and fat products | 22.2 | 44 | 18 | 26.3 | 26.9 | 21.1 | 38.8 | 17.2 | 25 | 24.9 | 22.2 | 50.6 | 15.7 | 28.6 | 25.2 | 0.92 | 0.7 | 0.783 |
| **Beverages** | 1373.20 | 684.7 | 1290.40 | 1456.00 | 1356.60 | 1444.60 | 697 | 1353.80 | 1535.50 | 1401.50 | 1568.30 | 695.8 | 1502.70 | 1634.00 | 1521.50 | **00021** | **00281** | **00083** |
| Water | 836.3 | 637.4 | 755.7 | 916.9 | 804 | 908.3 | 593 | 841.7 | 974.9 | 859.6 | 961 | 602.3 | 903.5 | 1018.40 | 917 | 00919 | 0214 | 0101 |
| Hot drinks | 379.6 | 325.2 | 350.7 | 408.5 | 411.1 | 443.9 | 354.5 | 400.5 | 487.3 | 464.3 | 521.1 | 401.6 | 484.2 | 558.1 | 538.3 | **<0.001** | **<0.001** | **<0.001** |
| Sweetened drinks | 157.3 | 261.9 | 123.1 | 191.6 | 141.5 | 92.5 | 278.2 | 56.7 | 128.3 | 77.7 | 86.2 | 188.9 | 64.2 | 108.3 | 66.2 | **<0.001** | **<0.001** | **<0.001** |
| **Fats and oils** | 25.7 | 27.4 | 22.7 | 28.8 | 29.9 | 24.3 | 20.1 | 22.1 | 26.4 | 26.7 | 28.5 | 21 | 26.3 | 30.7 | 29.9 | **00231** | 00579 | 00532 |
| Vegetal fats | 9.1 | 11.3 | 7.7 | 10.5 | 8.4 | 11 | 11.5 | 9.9 | 12.2 | 10 | 15.9 | 14.4 | 14.5 | 17.2 | 14.7 | **<0.001** | **<0.001** | **<0.001** |
| Animal fats | 8.8 | 15.9 | 7.1 | 10.6 | 10.9 | 8.2 | 11.9 | 6.9 | 9.5 | 9.5 | 8.4 | 11.5 | 7.4 | 9.5 | 9.1 | 0849 | 0249 | 019 |
| Sauces | 7.8 | 20 | 5.1 | 10.4 | 10.6 | 5 | 13.1 | 3.7 | 6.3 | 7.2 | 4.2 | 10.6 | 3.1 | 5.4 | 6.2 | 0057 | **00283** | **00223** |

Pval : pvalue of the General Linear Model to test the difference of intake by tertile of Medium/High food consumption ;

Pval1 : pvalue of the General Linear Model to test the difference of intake by tertile of Medium/High food consumption, adjusted on total energy intakes ;

Pval2 : pvalue of the General Linear Model model to test the difference of intake by tertile of Medium/High food consumption adjusted on total energy, income per consumption unit, socio-professional category, body mass index (BMI).

Mean adj, mean, adjusted on total energy (except for energy), income per consumption unit, socio-professional category, body mass index (BMI).

**Supplemental table 8.** Mean scores by tertile of Medium/High consumption among adults and adjusted means (on total energy intakes – except for energy IUC PCS and BMI);

| **Children (n=1775)** | | | | | | | | | | | | | | | | | | | |
| --- | --- | --- | --- | --- | --- | --- | --- | --- | --- | --- | --- | --- | --- | --- | --- | --- | --- | --- | --- |
|  | **T1 [098]** | | | | | | **T2 [98191]** | | | | | **T3 ≥191** | | | | |  |  |  |
|  | **Mean** | **Std** | **95%**  **LCL** | **95%**  **UCL** | **Mean**  **adj** | **Mean** | | **Std** | **95%**  **LCL** | **95%**  **UCL** | **Mean**  **adj** | **Mean** | **Std** | **95%**  **LCL** | **95%**  **UCL** | **Mean**  **adj** | **pval** | **pval_adj^1^** | **pval_adj^2^** |
| Total energy intake (kcal/d) | 1729.3 | 502.4 | 1669.7 | 1789.0 | 1578.4 | 1827.3 | | 657.4 | 1692.7 | 1961.9 | 1671.7 | 1942.1 | 559.8 | 1882.4 | 2001.8 | 1805.1 | **<0.001** | NA | **<0.001** |
| MAR* | 80.1 | 10.4 | 79.0 | 81.3 | 79.8 | 83.9 | | 8.1 | 82.8 | 84.9 | 82.7 | 86.0 | 5.9 | 85.5 | 86.5 | 84.0 | **<0.001** | **<0.001** | **<0.001** |
| MAR_2000 | 85.2 | 6.9 | 84.4 | 86.0 | 84.0 | 87.2 | | 5.5 | 86.6 | 87.8 | 86.4 | 87.2 | 5.9 | 86.5 | 87.8 | 87.0 | **<0.001** | **<0.001** | **<0.001** |
| MER** | 33.8 | 19.2 | 31.6 | 36.1 | 36.7 | 35.1 | | 19.7 | 31.8 | 38.4 | 36.2 | 33.8 | 24.6 | 31.0 | 36.6 | 32.7 | 0822 | **00238** | **0018** |
| SED (g/kcal) | 192.8 | 37.7 | 188.6 | 197.1 | 195.7 | 179.1 | | 28.7 | 175.6 | 182.6 | 180.3 | 162.6 | 27.5 | 159.8 | 165.4 | 161.2 | **<0.001** | **<0.001** | **<0.001** |
| sPNNS-GS2*** | 1.0 | 2.8 | 0.7 | 1.3 | 0.7 | 1.3 | | 2.7 | 0.9 | 1.6 | 1.1 | 1.6 | 3.0 | 1.3 | 2.0 | 1.6 | **<0.001** | **<0.001** | **<0.001** |
| **Adults (n=2121)** | | | | | | | | | | | | | | | | | | | |
|  | **T1 [0133]** | | | | | | **T2 [133248]** | | | | | **T3 ≥248** | | | | |  |  |  |
|  | **Mean** | **Std** | **95%**  **LCL** | **95%**  **UCL** | **Mean**  **adj** | **Mean** | | **Std** | **95%**  **LCL** | **95%**  **UCL** | **Mean**  **adj** | **Mean** | **Std** | **95%**  **LCL** | **95%**  **UCL** | **Mean**  **adj** | **pval** | **pval_adj^1^** | **pval_adj^2^** |
| Total energy intake (kcal/d) | 1830.7 | 697.5 | 1736.5 | 1924.9 | 1712.1 | 2045.1 | | 750.1 | 1948.6 | 2141.6 | 1935.0 | 2151.1 | 679.6 | 2080.7 | 2221.6 | 2041.9 | **<0.001** |  | **<0.001** |
| MAR | 72.7 | 11.8 | 71.1 | 74.3 | 72.8 | 79.0 | | 9.1 | 78.0 | 80.1 | 76.6 | 83.5 | 7.3 | 82.8 | 84.3 | 79.9 | **<0.001** | **<0.001** | **<0.001** |
| MAR_2000 | 78.6 | 7.6 | 77.7 | 79.5 | 76.7 | 81.3 | | 6.6 | 80.7 | 81.9 | 80.4 | 82.9 | 7.0 | 82.2 | 83.7 | 82.6 | **<0.001** | **<0.001** | **<0.001** |
| MER | 27.1 | 19.6 | 24.5 | 29.6 | 31.3 | 31.8 | | 22.7 | 29.3 | 34.4 | 31.8 | 30.1 | 21.0 | 27.9 | 32.2 | 27.9 | **00262** | **00012** | **00016** |
| SED (g/kcal) | 180.7 | 41.8 | 175.5 | 185.9 | 189.9 | 163.5 | | 38.1 | 159.1 | 167.8 | 167.9 | 147.5 | 33.1 | 144.0 | 151.0 | 149.6 | **<0.001** | **<0.001** | **<0.001** |
| sPNNS-GS2 | -1.2 | 3.6 | -1.6 | -0.8 | -1.8 | -0.9 | | 3.8 | -1.3 | -0.4 | -0.9 | -0.0 | 3.8 | -0.4 | 0.4 | 0.3 | **00012** | **<0.001** | **<0.001** |

*MAR Mean Adequacy Ratio

**MER Mean Excess Ratio

***sPNNS-GS2 Diet quality score developed nu National Program for Nutrition and Health

Pval : pvalue of the General Linear Model to test the difference of intake by tertile of Medium/High food consumption ;

Pval1 : pvalue of the General Linear Model to test the difference of intake by tertile of Medium/High food consumption, adjusted on total energy intakes ;

Pval2 : pvalue of the General Linear Model model to test the difference of intake by tertile of Medium/High food consumption adjusted on total energy, income per consumption unit, socio-professional category, body mass index (BMI).

Mean adj, mean, adjusted on total energy (except for energy), income per consumption unit, socio-professional category, body mass index (BMI).

**Supplemental table 9.** Percentage of adults and children meeting the minimal recommended (RV) value by tertile^$^ of Medium/High food consumption

|  | Children (n=1775) ^§^ | | | | Adults (n=2121) ^§^ | | | | |  |
| --- | --- | --- | --- | --- | --- | --- | --- | --- | --- | --- |
| Nutrient | T1  [0,98] | T2  [98,191] | T3  ≥191 | p-value | | T1 [0,133] | T2 [133,248] | T3  ≥248 | p-value | |
| Proteins (kg bw) | 77.6 | 89.9 | 87.9 | <0.001 | | 62.9 | 74.1 | 82.5 | <0.001 | |
| Proteins (% energy) | 99.1 | 99.8 | 99.4 | 0.361 | | 95.4 | 97.0 | 94.4 | 0.106 | |
| Carbohydrates | 97.3 | 94.5 | 95.5 | 0.264 | | 81.4 | 83 | 82.3 | 0.866 | |
| Fats | 22.0 | 19.5 | 15.8 | 0.249 | | 46.8 | 43.5 | 40.0 | 0.226 | |
| Linoleic acid | 13.7 | 8.6 | 11.7 | 0.117 | | 22.5 | 18.4 | 18.1 | 0.331 | |
| Alpha-linolenic acid | 0.9 | 0.2 | 0.6 | 0.478 | | 3.1 | 2.1 | 2.7 | 0.616 | |
| EPA | - no RV | - no RV | - no RV |  | | 10.4 | 12 | 16.7 | 0.0378 | |
| DHA | 16.8 | 21.1 | 18.5 | 0.399 | | 15.2 | 16.1 | 24.2 | 0.005 | |
| EPA+DHA | 12.4 | 16.9 | 15.3 | 0.293 | | 13.6 | 14.3 | 22.1 | 0.006 | |
| Fibers | 45.6 | 48.7 | 64.8 | <0.001 | | 4.5 | 6.8 | 13.3 | 0.003 | |
| Water | 13.3 | 17.2 | 31.7 | <0.001 | | 40.4 | 52.3 | 68.9 | <0.001 | |
| Calcium | 28.9 | 42.6 | 52.6 | <0.001 | | 24.5 | 43.2 | 55.4 | <0.001 | |
| Cupper | 34.3 | 35.7 | 52.7 | <0.001 | | 24.5 | 38.6 | 47.1 | <0.001 | |
| Iron low^‡^ | 56.2 | 68.1 | 68.8 | 0.0019 | | 26.3 | 33.7 | 39.1 | 0.005 | |
| Iron high^‡^ | 6.6 | 12.4 | 5.7 | 0.432 | | 0.7 | 1.2 | 3.2 | 0.008 | |
| Iodine | 54.8 | 64.4 | 72.5 | <0.001 | | 24 | 46 | 54.2 | <0.001 | |
| Magnesium | 30.0 | 38.3 | 53.6 | <0.001 | | 30.6 | 40.5 | 57.4 | <0.001 | |
| Phosphorus | 95.1 | 98.9 | 99.9 | <0.001 | | 91.5 | 98.6 | 99.2 | <0.001 | |
| Potassium | 50.5 | 65.9 | 68.8 | <0.001 | | 13.4 | 26.9 | 40.7 | <0.001 | |
| Selenium | 86.2 | 91.1 | 95.9 | 0.001 | | 84.1 | 90.3 | 96.7 | <0.001 | |
| Zinc^†^ | 25.6 | 44.7 | 44.5 | <0.001 | | 26.1 | 33.7 | 35.1 | <0.001 | |
| Sodium | 89.8 | 94.3 | 97.8 | <0.001 | | 87.8 | 95.5 | 97 | <0.001 | |
| Vitamin A | 49.9 | 63.4 | 73.7 | <0.001 | | 28.7 | 55.6 | 70.7 | <0.001 | |
| Vitamin B12 | 86.1 | 95.3 | 94.4 | <0.001 | | 43.4 | 58.6 | 59.4 | <0.001 | |
| Vitamin B1 | 89.7 | 94.0 | 97.3 | <0.001 | | 83.6 | 94.8 | 94.8 | <0.001 | |
| Vitamin B2 | 59.6 | 77.2 | 83.6 | <0.001 | | 31.1 | 52.6 | 68.9 | <0.001 | |
| Vitamin B3 | 70.8 | 67.1 | 67.9 | 0.532 | | 84.3 | 85.9 | 84.6 | 0.849 | |
| Vitamin B5 | 33.7 | 45.0 | 58.0 | <0.001 | | 27.1 | 43.4 | 61.0 | <0.001 | |
| Vitamin B6 | 63.3 | 77.2 | 80.2 | <0.001 | | 34.5 | 46.1 | 57.9 | <0.001 | |
| Vitamin B9 | 46.5 | 64.0 | 73.1 | <0.001 | | 20.9 | 34.3 | 54.1 | <0.001 | |
| Vitamin C | 54.1 | 64.0 | 74.5 | <0.001 | | 15.5 | 28.3 | 46 | <0.001 | |
| Vitamin D | 0.0 | 0.1 | 0.1 | 0.97 | | 0.4 | 0.0 | 0.7 | 0.0981 | |
| Vitamin E | 36.7 | 42.6 | 51.3 | 0.003 | | 34.9 | 40.8 | 58.2 | <0.001 | |

^$^Tertiles referred to low, middle and high consumption of Medium/High foods of live microbe content.

Values within [ ] show g/day.

^‡^ For adolescent girls (aged between 12 to 17 y. old) and women, French iron recommended values were available considering either light to moderate menstrual bleeding (iron low) or heavy menstrual bleeding (iron high). Percentage of individuals meeting the “iron low” minimal value were for the whole population (1775 children and 2121 adults), and percentage of individuals meeting the “iron high” minimal value were estimated among girls between 12 and 17 y. old (n = 459), and among women between 18 and 65 y. old (n = 1234).

^†^ In France, the daily value for zinc is depending on the assumed phytate content in the diet (300mg/d, 600mg/d or 900mg/d). In this table, the recommended values for zinc corresponds to 600mg/d (ANSES, 2021)

^§^ For “Iron high” only, n children =459 (girls between 12 and 17 y. old, and n adults = 1234 (women between 18 and 65 y. old) (ANSES, 2026b)
